# Group task-related component analysis (gTRCA): a multivariate method for inter-trial reproducibility and inter-subject similarity maximization for EEG data analysis

**DOI:** 10.1038/s41598-019-56962-2

**Published:** 2020-01-09

**Authors:** Hirokazu Tanaka

**Affiliations:** School of Information Science, Japan Advanced Institute of Science and Technology, 1-1 Asahidai, Nomi, Ishikawa 923-1292 Japan

**Keywords:** Dynamical systems, Neural decoding, Biomedical engineering

## Abstract

EEG is known to contain considerable inter-trial and inter-subject variability, which poses a challenge in any group-level EEG analyses. A true experimental effect must be reproducible even with variabilities in trials, sessions, and subjects. Extracting components that are reproducible across trials and subjects benefits both understanding common mechanisms in neural processing of cognitive functions and building robust brain-computer interfaces. This study extends our previous method (task-related component analysis, TRCA) by maximizing not only trial-by-trial reproducibility within single subjects but also similarity across a group of subjects, hence referred to as group TRCA (gTRCA). The problem of maximizing reproducibility of time series across trials and subjects is formulated as a generalized eigenvalue problem. We applied gTRCA to EEG data recorded from 35 subjects during a steady-state visual-evoked potential (SSVEP) experiment. The results revealed: (1) The group-representative data computed by gTRCA showed higher and consistent spectral peaks than other conventional methods; (2) Scalp maps obtained by gTRCA showed estimated source locations consistently within the occipital lobe; And (3) the high-dimensional features extracted by gTRCA are consistently mapped to a low-dimensional space. We conclude that gTRCA offers a framework for group-level EEG data analysis and brain-computer interfaces alternative in complement to grand averaging.

## Introduction

A major issue in subject-level and group-level EEG analysis is intra-subject and inter-subject variability across trials and sessions that originates from both endogenous factors and exogenous factors^1^. Endogenous factors of intra-subject variability include includes artifacts such as blinking, psychophysiological fluctuations, and fatigue, and exogenous factors include a temporal change in electrode impedance and positions. Inter-subject variability includes anatomical differences across subjects such as head shapes, skull conductivity, and patterns of brain gyrification (i.e., folding of the cerebral cortex), in which genetic differences play a primary role. These factors of variability consist of effects of non-interest and conceal an effect of interest related to a task. To sum, EEG is dynamic and non-stationary across sessions and subjects^2^, and the variability within and across subjects obfuscates both subject- and group-level analysis. Trial-mean across subjects (grand mean) is a simple and robust solution to improve signal-to-noise ratio by averaging out effects of non-interest within the group. In addition to univariate averaging, several multivariate methods have been proposed to enhance an experimental effect, including PCA-based ERP analysis^3–11^, partial-least-squares analysis^12^, independent component analysis^13,14^ (see more detail in Discussion).

Here, we propose a multivariate method to control non-reproducible components and enhance reproducible components across both trials and subjects. A real experimental effect in EEG data must be reproducible with controlled protocols even with single-trial variabilities and individual differences. A strong taking of this principle leads us to a premise that an experimental effect of interest can be defined as trial-reproducible patterns in the data within and across subjects. The principle of trial-reproducibility maximization was formulated as an algorithm named task-related component analysis (TRCA)^15–17^. The original TRCA was designed for a subject-level analysis and can thus address only within-subject data variability. There have been a few recent methods based on trial reproducibility of EEG features across trials^18^ or across subjects^19^, so maximizing reproducibility across trials and similarity across subjects points to a sensible direction to address intra- and inter-subject variability. The proposed method is an extension of TRCA to find a spatial filter that achieves trial reproducibility and similarity maximization across trials from a group of subjects, thereby referred to as group TRCA (gTRCA). We think it beneficial to extract reproducible components for the purposes of understanding a common mechanism in neural processing and of constructing robust brain-computer interfaces under variabilities inherent in EEG signals.

The problem of maximizing covariance across trials can be formulated as a generalized eigenvalue problem, and is efficiently solved by using standard numerical methods as proposed in our previous works on TRCA^15–17^. In the following sections, we will first show that gTRCA is formulated and solved efficiently as a generalized eigenvalue problem, and then test gTRCA with a publicly available EEG dataset of steady-state visual evoked potentials (SSVEPs) (Wang *et al*., 2016). We chose the SSVEP dataset because the issue of non-reproducible EEG data has been gathering attention in brain-computer interface (BCI) field. Besides, SSVEPs are known to require little training, to show little jitters in response latency, and to be robust among a subject group^20,21^, yet exhibit considerable intra- and inter-subject variability^22^. Thus, the SSVEP dataset provides an ideal test bed for the current study, with which we will show the algorithm provides a solution to address the intra- and across-subject non-stationarity problem in BCIs. Finally, we will demonstrate that gTRCA provides a predictive filter for a new subject with only one trial.

## Methods

### Task-related component analysis for single subject

**Figure 1:**
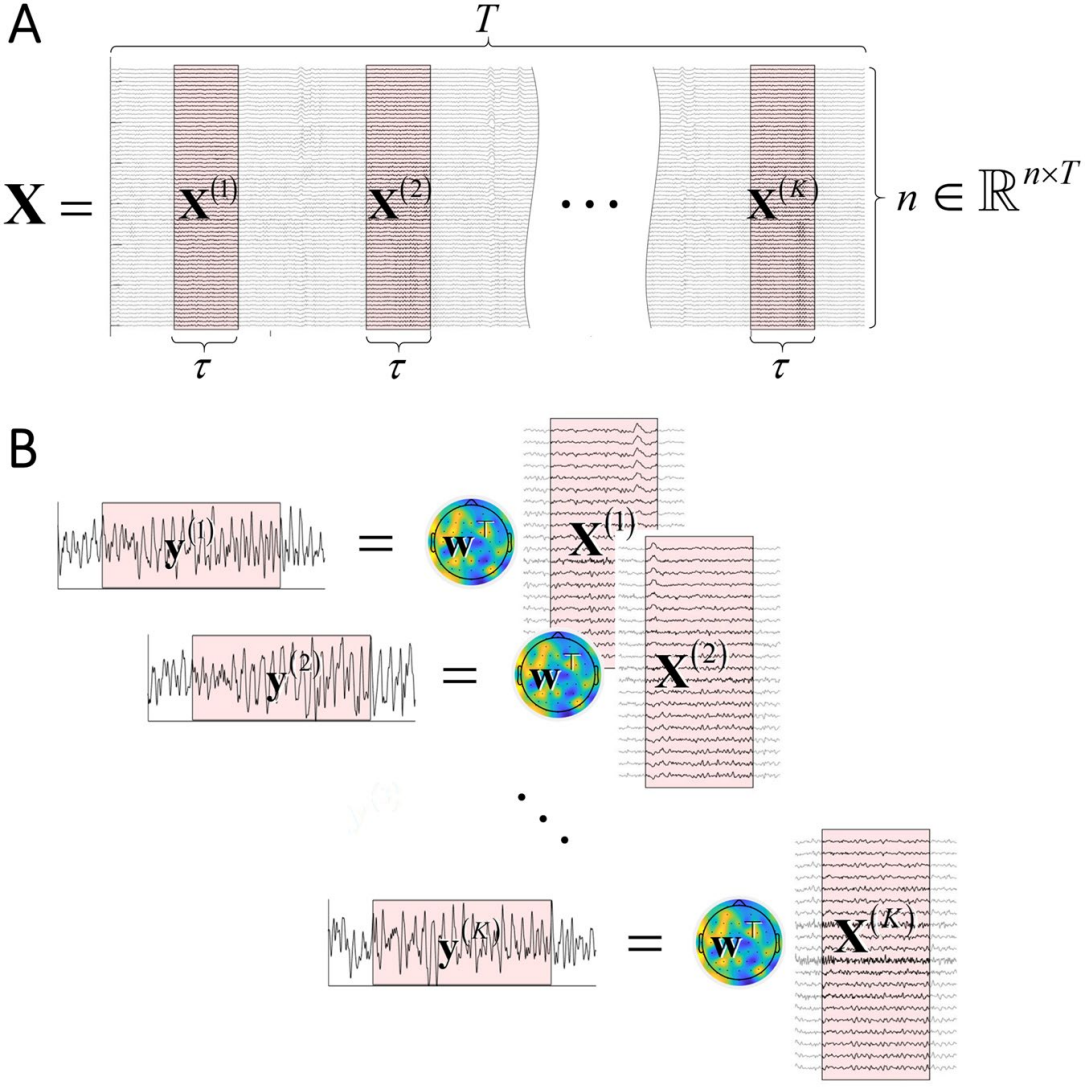
Schematic illustration of TRCA. (**A**) EEG data for a single subject is given in terms of *n* × *T* matrix **X** and *n* × *τ* time-windowed matrices ${{\bf{X}}}^{(k)}\,(k=1,\cdot \cdot \cdot ,K)$. The shaded areas denote the periods of *K* trials. (**B**) Trial-reproducible components (${{\bf{y}}}^{(k)}\,(k=1,\cdot \cdot \cdot ,K)$) as weighted sums of windowed matrices. The spatial filter **w** is determined so that *k*-th window activity **y**^(*k*)^ and $\ell$-th window activity ${{\bf{y}}}^{(\ell )}$ $(k\ne \ell )$ are maximally correlated.

Here we provide a brief review of TRCA for a single subject^15–17^. We note that TRCA is closely related to the xDAWN algorithm for enhancing evoked potentials^23^ and that in fact these two algorithms are equivalent to each other (see Supplementary Info). Figure 1 provides a schematic explanation of TRCA. Assume that we have *n*-channel data of length *T* in sample unit, denoted by ${\bf{X}}\in {{\mathbb{R}}}^{n\times T}$ (continuous data), and that there are *K* experimental trials with known event timing (i.e., latencies) and the time-windowed data of *k*-th trial is denoted by ${{\bf{X}}}^{(k)}\in {{\mathbb{R}}}^{n\times \tau }$ where *τ* is a trial length in sample unit (Fig. 1A). The timings of the trial windows may be specified by a user or determined by experimental timings such as those of stimulus presentation or subjects’ responses. To keep each channel within a fixed dynamic range, each channel data is normalized to zero mean and unit variance over the whole duration. Let us define the inter-trial cross-covariance as

1 $${\bf{S}}=\frac{1}{K(K-1)\tau }\mathop{\sum }\limits_{k\ne \ell }^{K}{{\bf{X}}}^{(k)}{{\bf{X}}}^{(\ell )\top }\in {{\mathbb{R}}}^{n\times n}$$

and the covariance matrix of continuous data as

2 $${\bf{Q}}=\frac{1}{T}{\bf{X}}{{\bf{X}}}^{\top }\in {{\mathbb{R}}}^{n\times n}$$

This definition of the matrix **S** contains *K*(*K*-1) summations. Note an equivalent but alternative definition of the matrix **S**,

3 $${\bf{S}}=\frac{K}{(K-1)\tau }({\bf{U}}{{\bf{U}}}^{\top }-\frac{1}{K}{\bf{V}})\in {{\mathbb{R}}}^{n\times n}$$

where the matrices **U** and **V** are defined as

4 $$\begin{array}{rcl}{\bf{U}} & = & \frac{1}{K}\mathop{\sum }\limits_{k=1}^{K}{{\bf{X}}}^{(k)}\in {{\mathbb{R}}}^{n\times \tau }\\ {\bf{V}} & = & \frac{1}{K}\mathop{\sum }\limits_{k=1}^{K}{{\bf{X}}}^{(k)}{{\bf{X}}}^{(k)\top }\in {{\mathbb{R}}}^{n\times n}\end{array}$$

This definition involves only *K* summations and accelerates the computation of the matrix S, especially when the number of trials *K* is large. Note that because of the $-\,\tfrac{1}{K}{\bf{V}}$ term in the parentheses, the matrix is not positive semidefinite hence can have negative eigenvalues.

Given the **S** and **Q** matrices, the problem of TRCA is now formulated as:

5 $${\rm{maximize}}\,{{\bf{w}}}^{\top }{\bf{S}}{\bf{w}}\,{\rm{subject}}\,{\rm{to}}\,{{\bf{w}}}^{\top }{\bf{Q}}{\bf{w}}=1$$

where ${\bf{w}}\in {{\rm{R}}}^{n}$ is a spatial filter that extracts a component maximally reproducible over trials. Once the filter **w** is obtained, the reproducible component is ${\bf{y}}={{\bf{w}}}^{\top }{\bf{X}}\in {{\mathbb{R}}}^{1\times T}$ and its *k*-th trial is ${{\bf{y}}}^{(k)}={{\bf{w}}}^{\top }{{\bf{X}}}^{(k)}\in {{\mathbb{R}}}^{1\times \tau }$ (Fig. 1B). These components are hereafter referred to as task-related components (TRCs). This filter guarantees that **y**^(*k*)^ of *k*-th trial and **y**^(*l*)^ of *l*-th trial are maximally correlated and reproducible. Equivalently, TRCA is formulated as a Rayleigh-Ritz eigenvalue problem as

6 $$\hat{{\bf{w}}}=\mathop{{\rm{argmax}}}\limits_{{\bf{w}}}\frac{{{\bf{w}}}^{\top }{\bf{S}}{\bf{w}}}{{{\bf{w}}}^{\top }{\bf{Q}}{\bf{w}}}$$

These are treated as a generalized eigendecomposition problem **Sw **= *λ***Qw**, so **w** may be obtained as eigenvectors of the matrix **Q**^−1^**S**. Note that, for the principal eigenvalue *λ* and eigenvector **w**, $$\lambda ={{\bf{w}}}^{\top }{\bf{S}}{\bf{w}}=\frac{1}{K(K-1)\tau }$$$$\mathop{\sum }\limits_{k\ne \ell }^{K}{{\bf{y}}}^{(k)}{{\bf{y}}}^{(\ell )\top }$$, so the eigenvalues are a measure of trial-to-trial reproducibility. Statistical significance of eigenvalues may be tested by a resampling test against a null hypothesis that there are no reproducible components for given experimental time windows^15^. The sign of the eigenvector is determined so that the reproducible component $${\bf{y}}={{\bf{w}}}^{\top }{\bf{X}}$$ has a positive projection onto the original data. Note that the reproducible component is unitless due to the normalization constraint $${\bf{y}}{{\bf{y}}}^{\top }=1$$. Once the filters are determined, corresponding spatial maps are constructed as detailed in^24^. If all of *N* eigenvectors are computed, *N* spatial maps corresponding to the eigenvectors are the columns of $${{\bf{W}}}^{-\top }$$ where $${\bf{W}}=(\begin{array}{ccc}{{\bf{w}}}_{1} & \cdots  & {{\bf{w}}}_{N}\end{array})\in {{\mathbb{R}}}^{n\times n}$$ is the eigenvector matrix. Here, we are interested in only a principal eigenvector **w**; in this case, the map is computed as $$\frac{1}{T}{\bf{X}}{{\bf{y}}}^{\top }=\frac{1}{T}{\bf{X}}{{\bf{X}}}^{\top }{\bf{w}}={\bf{Q}}{\bf{w}}$$. The data matrix **X** and the reproducible component **y** are unitless due to the normalization, so the corresponding map is also unitless.

Note that if we introduce ${\bf{v}}={{\bf{Q}}}^{\tfrac{1}{2}}{\bf{w}}$ and $\tilde{{\bf{S}}}={{\bf{Q}}}^{-\tfrac{1}{2}}{\bf{S}}{{\bf{Q}}}^{-\tfrac{1}{2}}$, the optimization problem becomes

7 $${\rm{maximize}}\,{{\bf{v}}}^{\top }\tilde{{\bf{S}}}{\bf{v}}\,{\rm{subject}}\,{\rm{to}}\,{{\bf{v}}}^{\top }{\bf{v}}=1$$

where ${{\bf{Q}}}^{\tfrac{1}{2}}$ is a symmetric matrix root of **Q**, hence $\tilde{{\bf{S}}}$ is a symmetric matrix. This is equivalent to formulation of principal component analysis (PCA), so various methods developed for PCA and its variants such as sparse PCA^25,26^, incremental PCA^27,28^, denoising source separation^29,30^, and joint decorrelation^31^ can be applied to TRCA, but we will not discusses them here.

### Group task-related component analysis (gTRCA)

**Figure 2:**
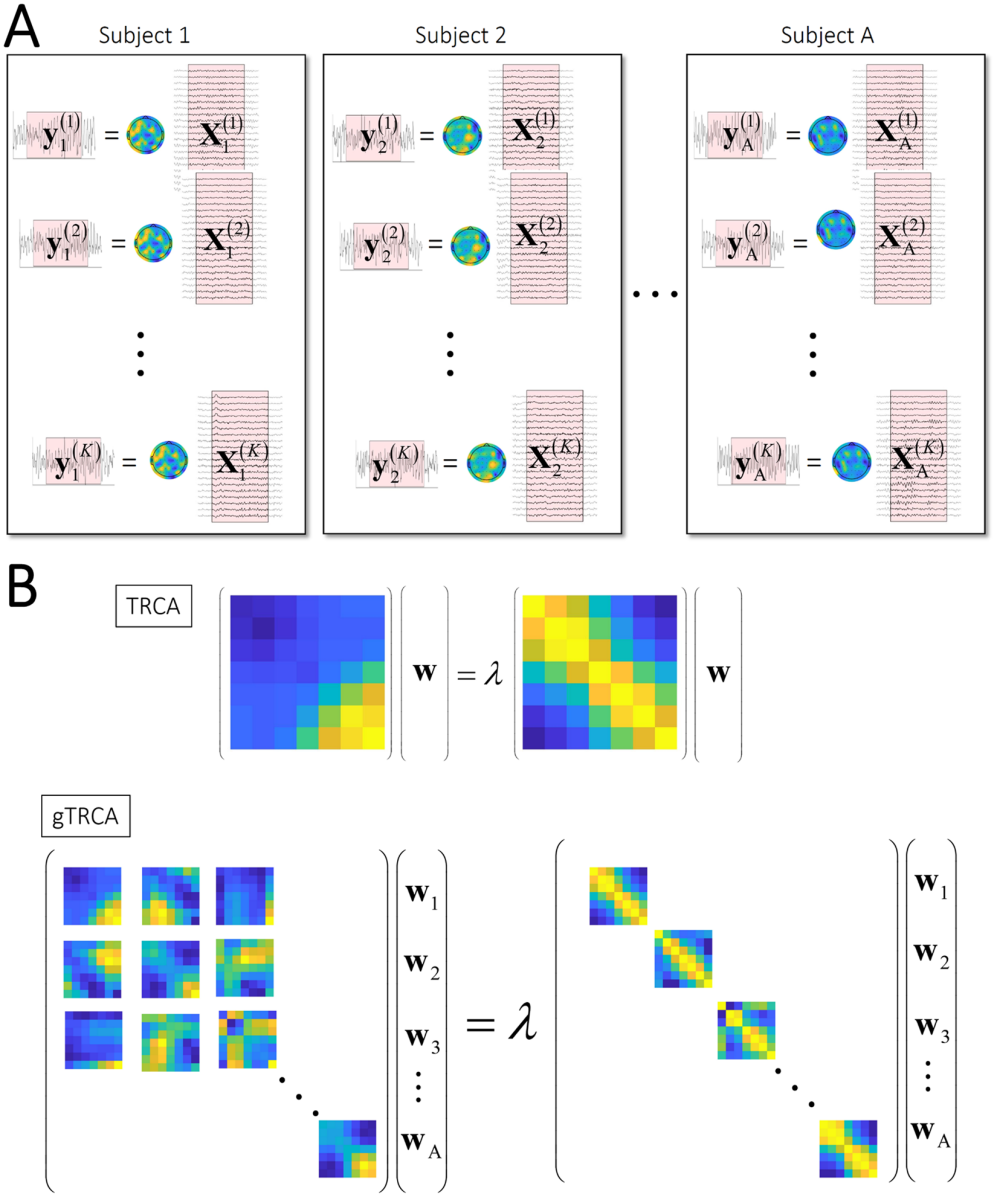
Schematic illustration of group TRCA (gTRCA). (**A**) gTRCA maximizes the covariances not only within individual subjects (${{\bf{y}}}_{\alpha }^{(k)}$ and ${{\bf{y}}}_{\alpha }^{(\ell )}$) but also across subjects (${{\bf{y}}}_{\alpha }^{(k)}$ and ${{\bf{y}}}_{\beta }^{(\ell )}$) by optimizing subject-specific spatial filters ${{\bf{w}}}_{\alpha }\,(\alpha =1,\cdots ,{\rm{{\rm A}}})$. (**B**) Generalized eigendecomposition problems of TRCA for *n* × *n* matrices (top) and gTRCA for *n*Α × *n*Α matrices (bottom).

TRCA in its basic formulation extracts reproducible components between trial timings *within* individual subjects. In order to more effectively suppress experimental effects of non-interest than just simple averaging across subjects, however, we naturally want to extend it to a group-level analysis as well to address the group-level non-stationarity in a unified approach. We extend TRCA so that it incorporates inter-subject covariance terms into the objective function. Subject specific filters are constructed to extract common components that maximize covariances across trials and subjects. Figure 2A illustrates the basic concept; the spatial filters ${{\bf{w}}}_{\alpha }\,(\alpha =1,\cdot \cdot \cdot ,{\rm{{\rm A}}})$ extract trial-reproducible components $\{{{\bf{y}}}_{\alpha }^{(k)}\}\,(\alpha =1,\cdot \cdot \cdot ,{\rm{{\rm A}}},\,k=1,\cdot \cdot \cdot ,K)$ that have maximal mutual covariances to each other.

Suppose A subjects performing *K* trials of a same task. Subject-specific filters ${\{{{\bf{w}}}_{\alpha }\in {{\mathbb{R}}}^{n}\}}_{\alpha =1}^{{\rm{{\rm A}}}}$ are assembled into a single vector as

8 $${\bf{w}}=(\begin{array}{c}{{\bf{w}}}_{1}\\ \vdots \\ {{\bf{w}}}_{\alpha }\\ \vdots \\ {{\bf{w}}}_{{\rm{{\rm A}}}}\end{array})\in {{\mathbb{R}}}^{n{\rm{{\rm A}}}}$$

Let us denote continuous EEG of *α*-th subject by ${{\bf{X}}}_{\alpha }\in {{\mathbb{R}}}^{n\times T}$ and time-windowed EEG at *k*-th trial of *α*-th subject ${{\bf{X}}}_{\alpha }^{(k)}\in {{\mathbb{R}}}^{n\times \tau }$. The numbers of channels (*n*), time points (*T*) and trials (*K*) are assumed to be the same for all subjects for notational simplicity; this simplifying assumption may be relaxed easily. Let us introduce an *n*A × *n*A matrix **S** as

9 $${\bf{S}}=(\begin{array}{ccccc}2{{\bf{S}}}_{11} & \cdots  & {{\bf{S}}}_{1\alpha } & \cdots  & {{\bf{S}}}_{1{\rm{{\rm A}}}}\\ \vdots  & \ddots  & \vdots  &  & \vdots \\ {{\bf{S}}}_{\alpha 1} & \cdots  & 2{{\bf{S}}}_{\alpha \alpha } & \cdots  & {{\bf{S}}}_{\alpha {\rm{{\rm A}}}}\\ \vdots  &  & \vdots  & \ddots  & \vdots \\ {{\bf{S}}}_{{\rm{{\rm A}}}1} & \cdots  & {{\bf{S}}}_{{\rm{{\rm A}}}\alpha } & \cdots  & 2{{\bf{S}}}_{{\rm{{\rm A}}}{\rm{{\rm A}}}}\end{array})\in {{\mathbb{R}}}^{n{\rm{{\rm A}}}\times n{\rm{{\rm A}}}}$$

whose off-diagonal *n* × *n* block matrices are defined for $\alpha \ne \beta$ as,

10 $${{\bf{S}}}_{\alpha \beta }=\frac{1}{{K}^{2}\tau }\mathop{\sum }\limits_{k,\ell =1}^{K}{{\bf{X}}}_{\alpha }^{(k)}{{\bf{X}}}_{\beta }^{(\ell )\top }\in {{\mathbb{R}}}^{n\times n}$$

and diagonal *n* × *n* block matrices are defined for *α* = *β* as,

11 $${{\bf{S}}}_{\alpha \alpha }=\frac{1}{K(K-1)\tau }\mathop{\sum }\limits_{k,\ell =1,k\ne \ell }^{K}{{\bf{X}}}_{\alpha }^{(k)}{{\bf{X}}}_{\alpha }^{(\ell )\top }\in {{\mathbb{R}}}^{n\times n}$$

The block-diagonal matrices ${{\bf{S}}}_{\alpha \alpha }\,(\alpha =1,\cdots ,{\rm{{\rm A}}})$ represent the trial-by-trial covariance within subjects, and the block-off-diagonal matrices ${{\bf{S}}}_{\alpha \beta }\,(\alpha =1,\cdots ,{\rm{{\rm A}}},\,\beta =1,\cdots ,{\rm{{\rm A}}},\,\alpha \ne \beta )$ represent the trial-by-trial covariance across subjects. Note that

12 $$\begin{array}{c}\frac{1}{2}{{\bf{w}}}^{\top }{\bf{S}}{\bf{w}}={{\bf{w}}}_{1}^{\top }{{\bf{S}}}_{11}{{\bf{w}}}_{1}+\cdots +{{\bf{w}}}_{{\rm{{\rm A}}}}^{\top }{{\bf{S}}}_{{\rm{{\rm A}}}{\rm{{\rm A}}}}{{\bf{w}}}_{{\rm{{\rm A}}}}+{{\bf{w}}}_{1}^{\top }{{\bf{S}}}_{12}{{\bf{w}}}_{2}+\cdots +{{\bf{w}}}_{{\rm{{\rm A}}}-1}^{\top }{{\bf{S}}}_{{\rm{{\rm A}}}-1,{\rm{{\rm A}}}}{{\bf{w}}}_{{\rm{{\rm A}}}}\\ \,\,\,\,\,=\mathop{\sum }\limits_{\alpha =1}^{{\rm{A}}}{{\bf{w}}}_{\alpha }^{\top }{{\bf{S}}}_{\alpha \alpha }{{\bf{w}}}_{\alpha }+\sum _{1\le \alpha \ne \beta \le {\rm{{\rm A}}}}{{\bf{w}}}_{\alpha }^{\top }{{\bf{S}}}_{\alpha \beta }{{\bf{w}}}_{\beta }\end{array}$$

Here, the terms in the first sum and in the second sum denote inter-trial reproducibility within subjects and inter-subject similarity across subjects, respectively. Hence, by maximizing ${{\bf{w}}}^{\top }{\bf{S}}{\bf{w}}$, the spatial filters extract common components across not only trials within subjects but also across subjects. Note that, if the off-diagonal block matrices ${{\bf{S}}}_{\alpha \beta }\,(\alpha \ne \beta )$ are set to zero (i.e., neglecting the inter-subject covariance), the objective function (12) is reduced to that of TRCA.

These matrices are efficiently computed as

13 $${{\bf{S}}}_{\alpha \beta }=\{\begin{array}{c}\frac{K}{(K-1)\tau }({{\bf{U}}}_{\alpha }{{\bf{U}}}_{\alpha }^{\top }-\frac{1}{K}{{\bf{V}}}_{\alpha })\,(\alpha =\beta )\\ \,\,\,\,\frac{1}{\tau }{{\bf{U}}}_{\alpha }{{\bf{U}}}_{\beta }^{\top }\,\,\,\,\,\,\,\,\,\,\,\,\,\,\,\,\,\,\,\,\,\,\,\,\,\,\,\,(\alpha \ne \beta )\end{array}$$

where

14 $${{\bf{U}}}_{\alpha }=\frac{1}{K}\mathop{\sum }\limits_{k=1}^{K}{{\bf{X}}}_{\alpha }^{(k)}$$

and

15 $${{\bf{V}}}_{\alpha }=\frac{1}{K}\mathop{\sum }\limits_{k=1}^{K}{{\bf{X}}}_{\alpha }^{(k)}{{\bf{X}}}_{\alpha }^{(k)\top }$$

Similarly, the full covariance matrix is defined as

16 $${\bf{Q}}={\rm{blkdiag}}({{\bf{Q}}}_{1},\cdots ,{{\bf{Q}}}_{\alpha },\cdots ,{{\bf{Q}}}_{{\rm{{\rm A}}}})\in {{\mathbb{R}}}^{n{\rm{{\rm A}}}\times n{\rm{{\rm A}}}}$$

where each block-diagonal matrix represents the covariance matrix of each subject,

17 $${{\bf{Q}}}_{\alpha }=\frac{1}{T}{{\bf{X}}}_{\alpha }{{\bf{X}}}_{\alpha }^{\top }\in {{\mathbb{R}}}^{n\times n}$$

As in the original TRCA formula, the filter **w** is obtained as the principal eigenvector of the matrix **Q**^−1^**S**. Figure 2B illustrates TRCA and gTRCA as general eigenvalue problems. The matrices **S** and **Q** are *n*A × *n*A matrices. The numbers of channels and subjects can be more than a hundred, resulting that $n{\rm{{\rm A}}}$ may be more than thousands, but standard numerical methods solve this high-dimensional eigenvalue problem. We used a Matlab implementation (i.e., eig(S, Q)).

As shown in our previous work, only a few TRCs are statistically significant in a resampling test. In the case of the SSVEP dataset described in this study, the largest eigenvalue was distinctly separated from the other eigenvalues (see Supplementary Info), so we here focus on only the principal eigenvector with the largest eigenvalue. Once the filters ${\{{{\bf{w}}}_{\alpha }\in {{\mathbb{R}}}^{n}\}}_{\alpha =1}^{{\rm{{\rm A}}}}$ are obtained, the TRC in *k*-th trial of *α*-th subject is ${{\bf{y}}}_{\alpha }^{(k)}={{\bf{w}}}_{\alpha }^{\top }{{\bf{X}}}_{\alpha }^{(k)}\in {{\mathbb{R}}}^{1\times \tau }$, and the mean TRC averaged over trials of *α*-th subject is ${{\bf{y}}}_{\alpha }=\frac{1}{K}\mathop{\sum }\limits_{k=1}^{K}{{\bf{y}}}_{\alpha }^{(k)}={{\bf{w}}}_{\alpha }^{\top }{{\bf{U}}}_{\alpha }\in {{\mathbb{R}}}^{1\times \tau }$. A corresponding scalp maps is computed as ${{\bf{Q}}}_{\alpha }{{\bf{w}}}_{\alpha }$ for *α*-th subject^24^.

### Predictive filter computed from group data

gTRCA formulated above requires data of multiple blocks for each subject to construct covariance matrices. When group data of A subjects is already available and then a single-trial data of a new subject is included, we would like a weight vector for the new subject from the existing group data. This scheme is considered in and known as zero-training BCI^32^ or semi-supervised learning^33^, which exploits the prior data as a supervising signal. We here propose to maximize the cross covariance between TRCs of A subjects and a TRC of a new subject.

Let us suppose that the full data of A subjects and the corresponding spatial filters are already computed using the method explained above. The problem is to construct a spatial filter of a new, (A + 1)-th subject when only data of one trial from this subject is available. Recall that the objective function of gTRCA (12) is rewritten as a sum of covariance between all possible TRCs from *K* trials of A subjects defined as

18 $$\mathop{\sum }\limits_{\alpha =1}^{{\rm{{\rm A}}}}\sum _{k\ne \ell }{{\bf{y}}}_{\alpha }^{(k)}{{\bf{y}}}_{\alpha }^{(\ell )\top }+\sum _{1\le \alpha  < \beta \le {\rm{{\rm A}}}}\sum _{k,\ell }{{\bf{y}}}_{\alpha }^{(k)}{{\bf{y}}}_{\beta }^{(\ell )\top }$$

Hence, when data from the first trial data ${{\bf{X}}}_{{\rm{{\rm A}}}+1}^{(1)}\in {{\rm{R}}}^{n\times \tau }$ and covariance ${{\bf{Q}}}_{{\rm{{\rm A}}}+1}\in {{\mathbb{R}}}^{n\times n}$ of the new subject are available, it is natural to require that a TRC (${{\bf{y}}}_{{\rm{{\rm A}}}+1}^{(1)}={{\bf{w}}}_{{\rm{{\rm A}}}+1}^{\top }{{\bf{X}}}_{{\rm{{\rm A}}}+1}^{(1)}\in {{\mathbb{R}}}^{1\times \tau }$) from the new subject be maximally correlated with all the TRCs ${\{{{\bf{y}}}_{\alpha }^{(k)}\}}_{\alpha =1,\cdots ,{\rm{{\rm A}}},k=1,\cdots ,K}$ by maximizing

19 $${{\bf{y}}}_{{\rm{{\rm A}}}+1}^{(1)}{(\frac{1}{K{\rm{{\rm A}}}}\mathop{\sum }\limits_{\alpha =1}^{{\rm{{\rm A}}}}\mathop{\sum }\limits_{k=1}^{K}{{\bf{y}}}_{\alpha }^{(k)})}^{\top }={{\bf{y}}}_{{\rm{{\rm A}}}+1}^{(1)}{(\frac{1}{{\rm{{\rm A}}}}\mathop{\sum }\limits_{\alpha =1}^{{\rm{{\rm A}}}}{{\bf{y}}}_{\alpha })}^{\top }$$

The spatial filter ${{\bf{w}}}_{{\rm{{\rm A}}}+1}$ for subject A + 1 is determined so that this objective function is maximized under a normalization constraint that ${{\bf{y}}}_{{\rm{{\rm A}}}+1}$ has a unit variance. This constrained optimization problem is formulated and solved by introducing an augmented Lagrangian function,

20 $$\frac{1}{{\rm{{\rm A}}}}\mathop{\sum }\limits_{\alpha =1}^{{\rm{{\rm A}}}}{{\bf{y}}}_{\alpha }{{\bf{y}}}_{{\rm{{\rm A}}}+1}^{(1)\top }-\lambda ({{\bf{w}}}_{{\rm{{\rm A}}}+1}^{\top }{{\bf{Q}}}_{{\rm{{\rm A}}}+1}{{\bf{w}}}_{{\rm{{\rm A}}}+1}-1)=\frac{1}{{\rm{{\rm A}}}}\mathop{\sum }\limits_{\alpha =1}^{{\rm{{\rm A}}}}{{\bf{w}}}_{\alpha }^{\top }{{\bf{U}}}_{\alpha }{{\bf{X}}}_{{\rm{{\rm A}}}+1}^{(1)\top }{{\bf{w}}}_{{\rm{{\rm A}}}+1}-\lambda ({{\bf{w}}}_{{\rm{{\rm A}}}+1}^{\top }{{\bf{Q}}}_{{\rm{{\rm A}}}+1}{{\bf{w}}}_{{\rm{{\rm A}}}+1}-1)$$

Here ${{\bf{U}}}_{\alpha }=\frac{1}{K}\mathop{\sum }\limits_{k=1}^{K}{{\bf{X}}}_{\alpha }^{(k)}\in {{\mathbb{R}}}^{n\times \tau }$. Then, the weight vector ${{\bf{w}}}_{{\rm{{\rm A}}}+1}$ is determined as a weighted sum of ${\{{{\bf{w}}}_{\alpha }\}}_{\alpha =1}^{{\rm{{\rm A}}}}$ as

21 $${{\bf{w}}}_{{\rm{{\rm A}}}+1}=\frac{1}{2{\rm{{\rm A}}}\lambda }{{\bf{Q}}}_{{\rm{{\rm A}}}+1}^{-1}{{\bf{X}}}_{{\rm{{\rm A}}}+1}^{(1)}\mathop{\sum }\limits_{\alpha =1}^{{\rm{{\rm A}}}}{{\bf{U}}}_{\alpha }^{\top }{{\bf{w}}}_{\alpha }$$

The Lagrange multiplier *λ* may be determined so that ${{\bf{y}}}_{{\rm{{\rm A}}}+1}={{\bf{w}}}_{{\rm{{\rm A}}}+1}^{\top }{{\bf{X}}}_{{\rm{{\rm A}}}+1}$ has a unit variance. The derived filter (21) has an intuitive interpretation; the weight vector ${{\bf{w}}}_{{\rm{{\rm A}}}+1}$ is a weighted sum of the existing filters ${\{{{\bf{w}}}_{\alpha }\}}_{\alpha =1}^{{\rm{{\rm A}}}}$ with coefficients ${{\bf{X}}}_{{\rm{{\rm A}}}+1}^{(1)}{{\bf{U}}}_{\alpha }^{\top }\in {{\mathbb{R}}}^{n\times n}$, which is a measure of similarity between the new subject and *α*-th subject. Simply put, the predictive filter exploits the similarity in EEG between the new subjects and the existing subjects, and places large weights on spatial filters **w**_α_ if the new data ${{\bf{X}}}_{{\rm{{\rm A}}}+1}^{(1)}$ resembles ${{\bf{U}}}_{\alpha }=\frac{1}{K}\mathop{\sum }\limits_{k=1}^{K}{{\bf{X}}}_{\alpha }^{(k)}$. In this way, the spatial filter of the new subject is predictively constructed in a way that it combines the existing filters of subjects with similar data. The performance of predictive filters was evaluated by comparing with the spatial filters constructed with the full dataset.

### gTRCA algorithm

The gTRCA algorithm is summarized as below:

Input: EEG data ${\{{{\bf{X}}}_{\alpha }\}}_{\alpha =1}^{{\rm{{\rm A}}}}$, trial onset timings {*t*_*k*_}, and trial duration *τ*.

1. Compute ${{\bf{U}}}_{\alpha }=\frac{1}{K}\mathop{\sum }\limits_{k=1}^{K}{{\bf{X}}}_{\alpha }^{(k)}$ and ${{\bf{V}}}_{\alpha }=\frac{1}{K}\mathop{\sum }\limits_{k=1}^{K}{{\bf{X}}}_{\alpha }^{(k)}{{\bf{X}}}_{\alpha }^{(k){\top }}$ for all subjects *α* = 1, …, A.
2. Compute $${{\bf{S}}}_{\alpha \beta }=\{\begin{array}{c}\frac{K}{(K-1)\tau }({{\bf{U}}}_{\alpha }{{\bf{U}}}_{\alpha }^{\top }-\frac{1}{K}{{\bf{V}}}_{\alpha })\,\,(\alpha =\beta )\\ \,\,\,\,\,\,\,\,\,\frac{1}{\tau }{{\bf{U}}}_{\alpha }{{\bf{U}}}_{\beta }^{\top }\,\,\,\,\,\,\,\,\,\,\,\,\,(\alpha \ne \beta )\end{array}$$ for all *α* and *β*.
3. Compute ${{\bf{Q}}}_{\alpha }=\frac{1}{T}{{\bf{X}}}_{\alpha }{{\bf{X}}}_{\alpha }^{\top }$ for all *α*= 1, …, A.
4. Compute the dominant eigenvalue and eigenvector ${\bf{w}}={({{\bf{w}}}_{1}^{\top }\cdots {{\bf{w}}}_{\alpha }^{\top }\cdots {{\bf{w}}}_{{\rm{{\rm A}}}}^{\top })}^{\top }\in {{\rm{R}}}^{n{\rm{{\rm A}}}}$ of the generalized eigenvalue problem of the matrices **S** and **Q**.
  Output: spatial filters ${\{{{\bf{w}}}_{\alpha }\in {{\mathbb{R}}}^{n}\}}_{\alpha =1}^{{\rm{{\rm A}}}}$ and corresponding spatial maps ${\{{{\bf{Q}}}_{\alpha }{{\bf{w}}}_{\alpha }\in {{\mathbb{R}}}^{n}\}}_{\alpha =1}^{{\rm{{\rm A}}}}$ for subject 1 to subject Α.
  Additionally, if data (${{\bf{X}}}_{{\rm{{\rm A}}}+1}^{(1)}$ and ${{\bf{X}}}_{{\rm{{\rm A}}}+1}$) from a new subject is available, a few more steps are needed in which the predictive filter and the predicted condition are computed.
5. Compute ${{\bf{w}}}_{{\rm{{\rm A}}}+1}^{c}=\frac{1}{2{\rm{{\rm A}}}\lambda }{{\bf{Q}}}_{{\rm{{\rm A}}}+1}^{-1}{{\bf{X}}}_{{\rm{{\rm A}}}+1}^{(1)}\mathop{\sum }\limits_{\alpha =1}^{{\rm{{\rm A}}}}{{\bf{U}}}_{\alpha }^{c{\top }}{{\bf{w}}}_{\alpha }^{c}$ for all conditions.
6. Compute correlation coefficients between ${{\bf{y}}}_{{\rm{{\rm A}}}+1}^{c}={{\bf{w}}}_{{\rm{{\rm A}}}+1}^{c\top }{{\bf{X}}}_{{\rm{{\rm A}}}+1}^{(1)}$ and $\frac{1}{{\rm{{\rm A}}}}\mathop{\sum }\limits_{\alpha =1}^{{\rm{{\rm A}}}}{{\bf{y}}}_{\alpha }^{c}$ for all conditions.

Output: predictive spatial filter **w**_A+1_, and corresponding spatial map **Q**_A+1_**w**_A+1_.

Example codes of Matlab are accompanied as Supplementary Data.

### t-SNE visualization of task-related components

Task-related components ${\{{{\bf{y}}}_{\alpha }^{(k)}\}}_{\alpha =1,\cdots ,{\rm{{\rm A}}},k=1,\cdots ,K}$ represent *τ* dimensional vectors (*τ* is a sampling length in a trial, typically ranging from hundreds to thousands), so their similarity (or dissimilarity) is difficult to visualize. We here employ t-distributed stochastic neighbor embedding (t-SNE), a visualization method of high-dimensional data by mapping original data in high dimensions onto low dimensions (typically two or three)^34^. Specifically, the t-SNE algorithm maps each *τ*-dimensional task-related component to a low-dimensional vector so that the distribution of *τ*-dimensional components is maximally preserved in the distribution of low-dimensional vectors in the sense of Kullback-Leiber divergence. The Matlab implementation of t-SNE was used with the default set of parameters (in particular, the value of perplexity was set to 30). The single-trial time series were mapped to the two-dimensional space, and the degree to which these two-dimensional points formed clusters was evaluated by calculating Fisher’s discriminant ratio (FDR), a measure of cluster separation^35^. A large value of FDR indicates that data points are closely aggregated within clusters and that one cluster is well separated to adjacent clusters.

### SSVEP data set

A publicly available dataset of steady-state visual evoked potentials was used^36^. The dataset contains EEG data from 35 subjects and 40 stimulation conditions that are characterized by stimulation frequencies and phases (frequencies ranging from 8 Hz to 15.8 Hz with an interval of 0.2 Hz, with phase difference of π/2 between adjacent frequencies), referred to as a joint frequency and phase modulation (JFPM) coding^37^. Each condition was repeated six times (therefore, six trials). 64-channel EEG was measured at the sampling rate of 250 Hz from −0.5 s to 5.5 s, and the stimulus was presented from 0 s to 5 s. A full account of and the web link to the dataset are found in Wang, *et al*.^36^. For the analyses, the stimulus presentation epoch (from 0 s to 5 s) was used. In terms of our notation, *n* = 64, *τ* = 1250, *K* = 6, *C* = 40, and Α = 35. For the interpretation, the conditions were reordered so that frequencies increased with the condition number (i.e., condition 1 with 8 Hz, and condition 40 with 15.8 Hz; adjacent conditions separated by 0.2 Hz in frequency and 90° in phase). Therefore, each stimulation condition was uniquely specified by its frequency. This dataset provides only time-windowed data (${{\bf{X}}}_{\alpha }^{c(k)}$ for *k*-th trial of condition *c* from *α*-th subject in our notation), and continuous data (${{\bf{X}}}_{\alpha }^{c}$ for condition *c* from *α*-th subject in our notation) were constructed by concatenating the time-windowed data horizontally.

First, gTRCA was applied separately to each condition as described in the section *gTRCA algorithm*; 40 spatial filters corresponding to 40 stimulation conditions for each subject were constructed. To remove baseline fluctuations and high-frequency noise, all scalp channel signals were bandpass-filtered with cutoff frequencies with 0.5 Hz and 49.5 Hz (FIR, Hamming windowed, transition bandwidth 1 Hz) before applying gTRCA. The onset of stimulus presentation served as a trial onset, and the trial duration *τ* was set to be 5 s, which was the period of the stimulus duration. The sign of the weight vector from each subject was determined so that the reproducible component was positively correlated with the EEG time courses of O1, O2 and Oz. To visualize the results, all the time series computed in one condition were averaged across 6 trials and 35 subjects, and the power spectral densities were computed from single-trial time series and then averaged. To observe the similarity between time series, 40 × 40 correlation matrix was computed by taking correlations between time series from different conditions. Also, the power spectral densities (PSDs) of these time series were computed in the Fourier domain. To quantify how these PSDs captured the stimulus frequency, a signal-to-noise ratio,

22 $$\frac{\text{PSD}({f}_{{\rm{stim}}})}{{\sum }_{f}{\rm{PSD}}(f)}$$

was computed for each condition, where *f*_stim_ is a stimulus frequency.

Next, a filter of a subject was constructed using the method described in *Predictive filter computed from group data*. We took a leave-one-out approach; α-th subject was removed from the full data set, and filters were constructed for the rest of 34 subjects using the method described in *Group trial-reproducible component analysis*. Then, given unlabeled data of first trial of subject α (${{\bf{X}}}_{\alpha }^{(1)}$) and the covariance (**Q**_*α*_), the filter for this subject was constructed as a weighted sum of the other subjects’ filters as described in *Predictive filter computed from group data*. To evaluate the performance of transfer learning, the time courses computed in the leave-one-out way were compared with those computed using the full dataset. Finally, the correlation coefficient between the time course of the new subject (**y**_A+1_) and the average time course from the other subjects ($\frac{1}{{\rm{{\rm A}}}}\mathop{\sum }\limits_{\alpha =1}^{{\rm{{\rm A}}}}{{\bf{y}}}_{\alpha }$) was computed for all condition as in *Prediction of experimental condition using predictive filter*. The condition with maximal value of correlation coefficient was chosen as a predicted condition. The above procedure was repeated for all subjects.

### Comparison with single-channel data and spatio-spectral decomposition (SSD)

To evaluate the performance of gTRCA algorithm, single-channel data was analyzed. Previous studies on SSVEPs have demonstrated that SSVEPs are best observed in occipital electrodes, so we chose the electrode Oz for single-channel data analysis^38^. Time-windowed data were averaged in the temporal domain over all trials and subjects to evaluate their mean and variance. In addition, time-windowed data were Fourier-transformed, and the corresponding PSDs were computed on a trial-by-trial basis, and then the mean and the variance of spectral densities were computed. The signal-to-noise ratio of PSDs defined in Eq. (22) was computed.

We also applied a spatial filter, spatio-spectral decomposition (SSD)^39^, which was designed to extract oscillatory components from EEG data. SSD is a spatial-filter method that maximizes the power of desired (i.e., user-specified) frequency band while suppressing the power of neighboring frequency bands. In order to apply SSD, a priori knowledge about the desired frequency band and its neighboring frequency bands must be supplied. The signal part was obtained by band-pass filtering data in the range of $[{f}_{{\rm{stim}}}-1,{f}_{{\rm{stim}}}+1]$ Hz, and the noise part was obtained by band-pass filtering data in the range of 2–30 Hz and them performing band-stop filtering in the range of $[{f}_{{\rm{stim}}}-3,{f}_{{\rm{stim}}}+3]$ Hz. Here *f*_stim_ denotes the stimulation frequency. SSD was applied on a subject basis, and the dominant component with the largest eigenvalue was analyzed further. Then, as in the single-electrode analysis, a grand mean in the temporal domain and the PSDs in the Fourier domain were computed.

### Spatial reproducibility of scalp maps

gTRCA and SSD return corresponding scalp maps. Spatial reproducibility of gTRCA and SSD was evaluated by correlation coefficients of scalp maps. The reproducibility of scalp maps was evaluated by computing the average of pair-wise correlation coefficients between scalp maps of all subjects in a given condition and between scalp maps of all conditions in a given subject; high values of the correlation coefficients suggest high reproducibility of the scalp maps or vice versa.

### Resampling-based statistical test

gTRCA optimizes a large number of filters (*n* channels × A subjects), so it is possible to overfit to the data and produce an artifactual component which is not present in the data. An appropriate statistical test is necessary before concluding a task-related component. We employ a resampling-based statistical test that has been introduced and validated in our previous studies^15–17^. The underlying assumption of TRCA is that there are certain reproducible components time-locked to the trial onsets. Therefore, an appropriate null hypothesis that there is no reproducible component time-locked to the trial onsets. The eigenvalues computed with the actual trial onsets are compared with eigenvalues computed with randomized trial onsets (see subsection 2.2 of Supplementary Info).

## Results

The proposed method, group TRCA, was applied to the dataset of SSVEP experiment in comparison with single-channel data and SSD. First, the reproducibility of time series was evaluated both in the time and frequency domains, and the corresponding scalp maps were compared for gTRCA and SSD. Second, the high-dimensional features of TRCs were mapped into the two-dimensional space by applying t-SNE to investigate the distributions of TRCs. Finally, the predictive filters for a new subject were computed and evaluated.

### Inter-subject similarity maximization

Here we report that gTRCA enhanced the trial reproducibility and the frequency responses in comparison with single-channel EEG recorded at Oz and spatio-spectral decomposition (SSD). The matrices **S** and **Q** for gTRCA (Eqs. (9) and (16)) were 2,240 dimensional (64 electrodes × 35 subjects), and its dominant eigenvalue and eigenvector were obtained efficiently as described in Methods. The matrix **S** was full-rank for all the conditions, so there was no numerical instability in computing the eigenvalues. For all the conditions, there was a gap between the first dominant and the second dominant eigenvalues (see subsection 2.1 of Supplementary Info). Since there is no rotational indeterminacy in gTRCA, the decomposition into components is unique (see Discussion). The distribution of eigenvalues suggests only one dominant component, and hence only the first dominant component in each condition was subjected to the following analyses.

The resampling-based test demonstrated that the dominant eigenvalue was statistically significant for all the conditions (see subsection 2.2 of Supplementary Info). We also tested how a preprocessing of dimensional reduction influenced the performance of gTRCA. Specifically, individual data was analyzed by applying PCA, and in most cases, 30 PCs were sufficient to explain about 90% of the original variance. The original data of individual subjects from 64 channels were hence reduced to 30 PCs. Subsequently, gTRCA was applied to the dimensionally reduced data. The result using the original data and the result using the dimensionally reduced data were mostly indistinguishable. Therefore, for the case of the SSVEP dataset, the effect of dimensional reduction did not significantly affect the performance of gTRCA (see subsection 2.3 of Supplementary Info).

**Figure 3:**
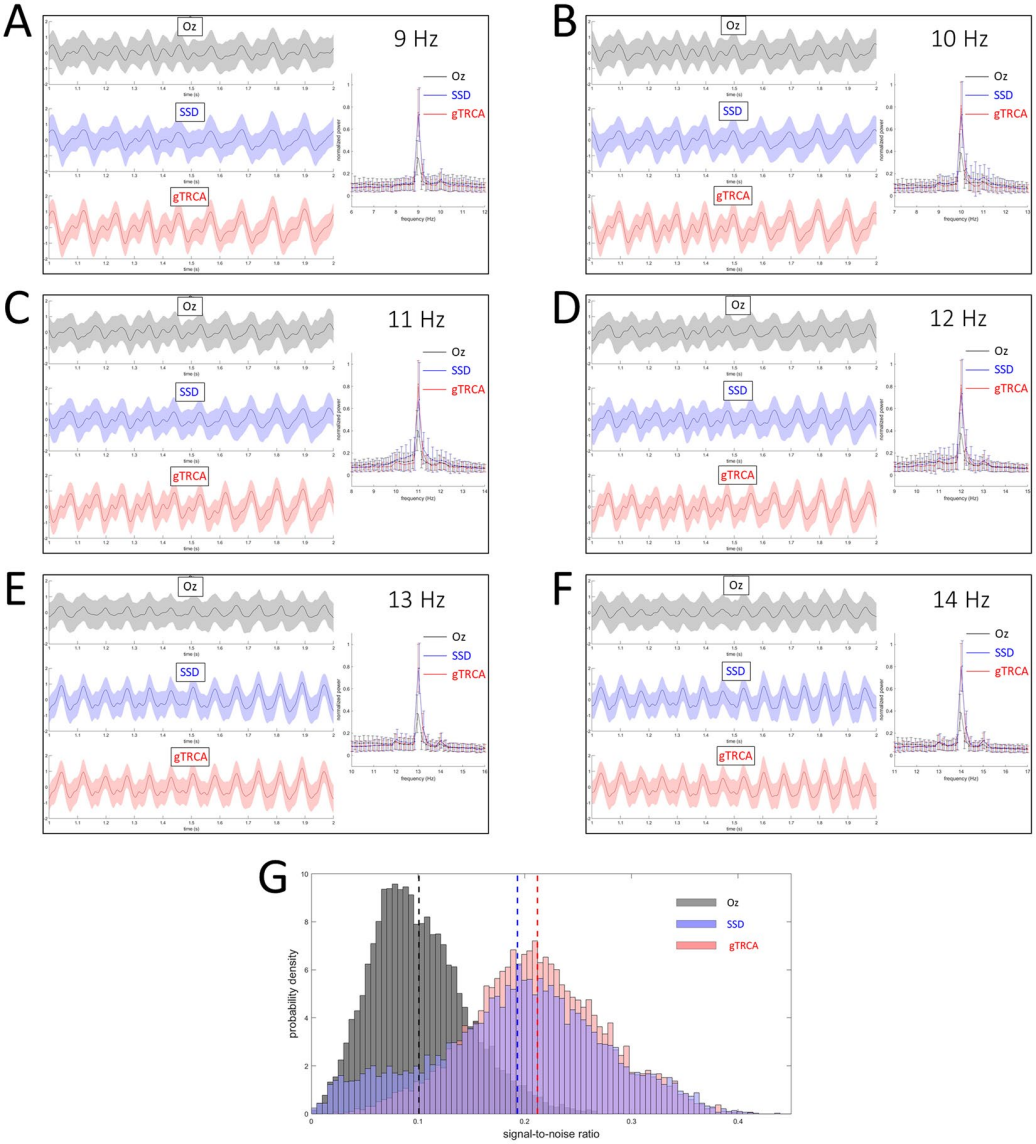
Comparison of EEG from a single electrode (Oz), spatio-spectral decomposition (SSD), and trial-reproducible components (gTRCA). (**A–F**) Six conditions (frequencies ranging from 9 Hz to 14 Hz with an interval of 1 Hz) out of 40 conditions are shown. In each panel, time courses of Oz (top, black lines) SSD components (middle, blue lines), and trial-reproducible components (bottom, red lines) averaged over trials and subjects are shown with standard deviations (shaded areas) from 1 to 2 seconds. Power-spectrum densities are plotted for Oz (black), SSD (blue), trial-reproducible components (red). (**B**) Histograms of signal-to-noise ratio computed from the single electrode (black bins), SSD (blue bins), and gTRCA (red bins). Dashed lines denote the mean values of Oz, SSD, and gTRCA, respectively.

Figure 3 summarizes the results. All the results from single-channel EEG, SSD and gTRCA exhibited the peaks at the frequencies of visual stimulation, indicating that SSVEP was entrained with the visual flickers (Fig. 3A–F). We observed that the TRCs were entrained more strongly and consistently than the single-channel EEG or SSD, and that the standard deviation of TRCs in the time domain was much smaller than that of single-channel EEG or SSD (0.953 for Oz, 0.859 for SSD, and 0.789 for gTRCA). There were statistically significant differences between the standard deviations of the three methods (unpaired t-test; between Oz and SSD, *t*(92075) = 223.6, *p* < 0.001; between Oz and gTRCA, *t*(99519) = 444.5, *p* < 0.001; and between SSD and gTRCA, *t*(95031) = 159.6, *p* < 0.001; Bonferroni corrected). Each PSD computed from TRCs in one condition demonstrated a peak that stood out from the other peaks computed from Oz or SSD. To study how concentrated the PSDs were around the stimulus frequencies, signal-to-noise ratios (Eq. (22)) were computed (Fig. 3G). Clearly, the signal-to-noise ratios of TRCs (mean 0.21 ± 0.063 (SD)) were statistically higher than those of single electrode (mean 0.10 ± 0.045 (SD)) or SSD (mean 0.19 ± 0.080 (SD)) (unpaired t-test; between Oz and SSD, *t*(16798) = 91.8, *p* < 0.001; between Oz and gTRCA, *t*(16798) = 131.4, *p* < 0.001; and between SSD and gTRCA, *t*(16798) = 16.7, *p* < 0.001; Bonferroni corrected). Although SSD captured the stimulation frequency in most trials, there were the considerable number of trials in which SSD failed as observed in the lower tail of the distribution (see the blue histogram in Fig. 3G). In summary, gTRCA enhanced the reproducibility of TRCs in both the temporal and frequency domains across trials and subjects.

**Figure 4:**
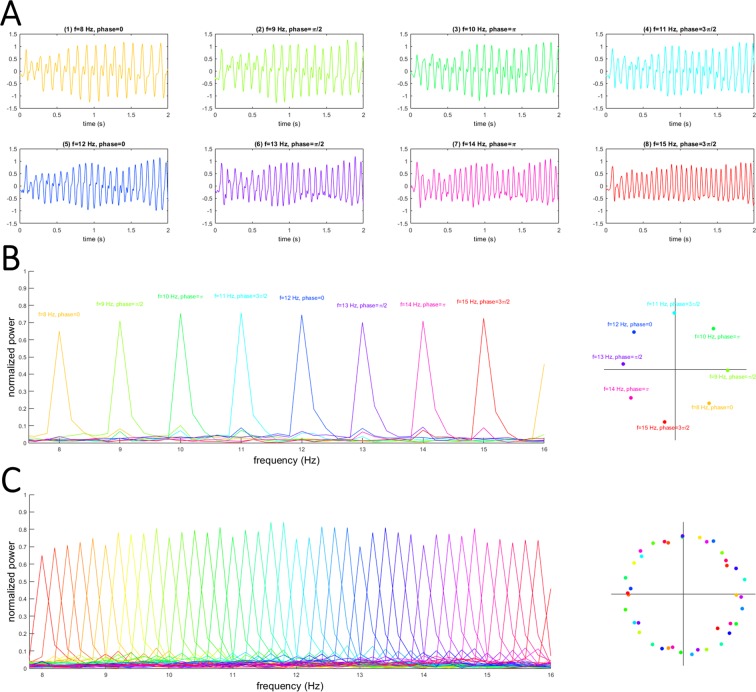
Trial-reproducible components and corresponding Fourier spectra. (**A**) Average time series of trial-reproducible components for the 8–15-Hz conditions. (**B**) Power spectral densities and Fourier phases of trial-reproducible components in Panel (A). (**C**) Power spectral densities and Fourier phases of all 40 conditions.

For a close inspection of the results of gTRCA, we plotted the average time series in responses to visual frequencies from 8 Hz to 15 Hz in steps of 1 Hz (Fig. 4A). The SSVEP TRCs obtained for 8 Hz to 11 Hz stimulation obviously showed bimodal waveforms, indicating contributions from higher harmonics; in contrast, the SSVEP TRCs obtained for 14 Hz and 15 Hz stimulation were unimodal in waveforms, indicating less contributions from higher harmonics. Fourier transforms separated these TRCs not only for their PSDs but also their phases (Fig. 4C), which clearly revealed that the signal had a structure designed for the joint frequency-phase modulation (JFPM) coding. Additionally, we analyzed the Fourier transform of TRCs from all of 40 stimulation conditions (Fig. 4C). The peaks of TRCs were clearly separated even though the intervals of stimulation frequencies were as small as 0.2 Hz. These results confirmed that gTRCA could extract the components that reflected the temporal and spectral features of visual flickering stimuli.

**Figure 5:**
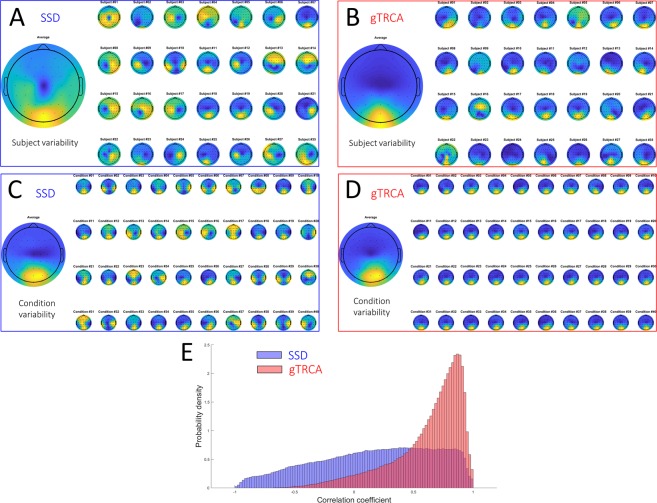
Scalp maps obtained by SSD and gTRCA. Scalp maps for all 35 subjects in one stimulation condition (9 Hz) obtained by (**A**) SSD and (**B**) gTRCA. Scalp maps of one representative subjects (Subject #10) for all 40 stimulation conditions obtained by (**C**) SSD and (**D**) gTRCA. On the left of Panels (**A–D**), average maps are shown. (**E**) Histograms of correlation coefficients of all pairs of scalp maps obtained by SSD (blue) and gTRCA (red).

Next, we examined the spatial reproducibility of scalp maps obtained by SSD and gTRCA. Figure 5 summarizes the scalp maps of all subjects in one condition (Fig. 5A (SSD) and 5B (gTRCA)) and the scalp maps of all conditions in one subject (Fig. 5C (SSD) and 5D (gTRCA)). The scalp maps obtained by SSD varied considerably across subjects in one condition (Fig. 5A) and across conditions within a subject (Fig. 5C). On the other hand, the scalp maps obtained by gTRCA showed consistent distribution centered at the occipital area near electrode Oz for all subjects both in one condition (Fig. 5B) as well all conditions one subject (Fig. 5D), consistent with previous studies reporting that SSVEPs are the most prominent in electrodes in the occipital area^21,40^. The correlation coefficients between the scalp maps of gTRCA (mean 0.62, SD 0.30) are much higher than those between the scalp maps of SSD (mean 0.17, SD 0.50) (Fig. 5E) (unpaired t-test, *t*(979299) = 813.4, *p* < 0.001), proving that the scalp maps of gTRCA were more reproducible across subjects and conditions than those of SSD. It is worth emphasizing that the improvement of reproducibility in scalp maps, across subjects and conditions, was achieved by gTRCA’s maximizing the reproducibility in the time domain. We note, however, that the variability found in the scalp maps of SSD might have reflected the true individual variability of source locations. To sum, we observed that gTRCA could capture the temporal and spatial characteristics of SSVEPs in a robust and reproducible manner, thereby demonstrating potential usefulness of the algorithm.

### t-SNE visualization

**Figure 6:**
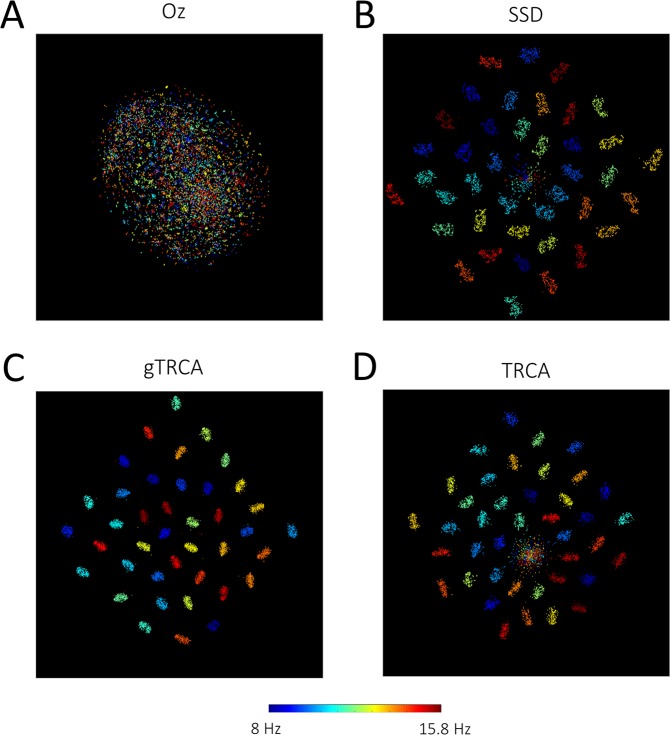
t-SNE visualization of single-trial data of (**A**) single channel (Oz), (**B**) SSD, (**C**) gTRCA, and (**D**) TRCA. Each two-dimensional point corresponds to a time series of a single trial colored by a stimulation condition (red and blue correspond 8 Hz and 15.8 Hz, respectively. See the color bar at the bottom). Note that a pair of trials from adjacent stimulation frequencies are not necessarily mapped closely to the two-dimensional space because two stimulation conditions of adjacent frequencies are separated by a 90° phase difference.

To further investigate reproducibility of SSVEP responses, t-SNE was employed to map original high-dimensional data into a low-dimensional space^34^. Single-trial time series extracted from the single electrode (Oz), by SSD or gTRCA had *τ*(=1250) dimensions, which were mapped to two-dimensional points by applying t-SNE. Figure 6 illustrates the results of t-SNE mapping. The two-dimensional points mapped from single-trial time series at the electrode Oz were scattered without any obvious cluster structures, thereby reflecting considerable intra- and inter-subject variability of SSVEP responses (Fig. 6A). The points mapped from the single-trial time series extracted by SSD were better clustered by the stimulation conditions, but there were some outlier data points around the origin that did not appear to belong to any cluster (Fig. 6B). In contrast, the two-dimensional points obtained by gTRCA formed clusters almost perfectly categorized by the stimulation conditions (Fig. 6C). These points were more tightly aggregated within the clusters, and the clusters were more separated to each other than those obtained by SSD. To further investigate the mechanism of gTRCA, we also applied TRCA (i.e., setting the off-diagonal block matrices ${{\boldsymbol{S}}}_{\alpha \beta }\,(\alpha \ne \beta )$ in Eq. (12). to zero, thereby neglecting inter-subject similarity). The two-dimensional points were clustered to some degree but not as tightly as those of gTRCA (Fig. 6D), indicating that the inter-subject terms played an essential role to extract the task-related components common to the subjects. These observations were quantified by computing Fisher’s discriminant ratio (FDR); 0.021 for the single-channel data, 0.59 for SSD, 0.49 for TRCA, and 1.42 for gTRCA. Therefore, inter-trial reproducibility and inter-subject similarity maximization play a key role in extracting the common feature from the EEG data.

### Predictive filter computed from group data

**Figure 7:**
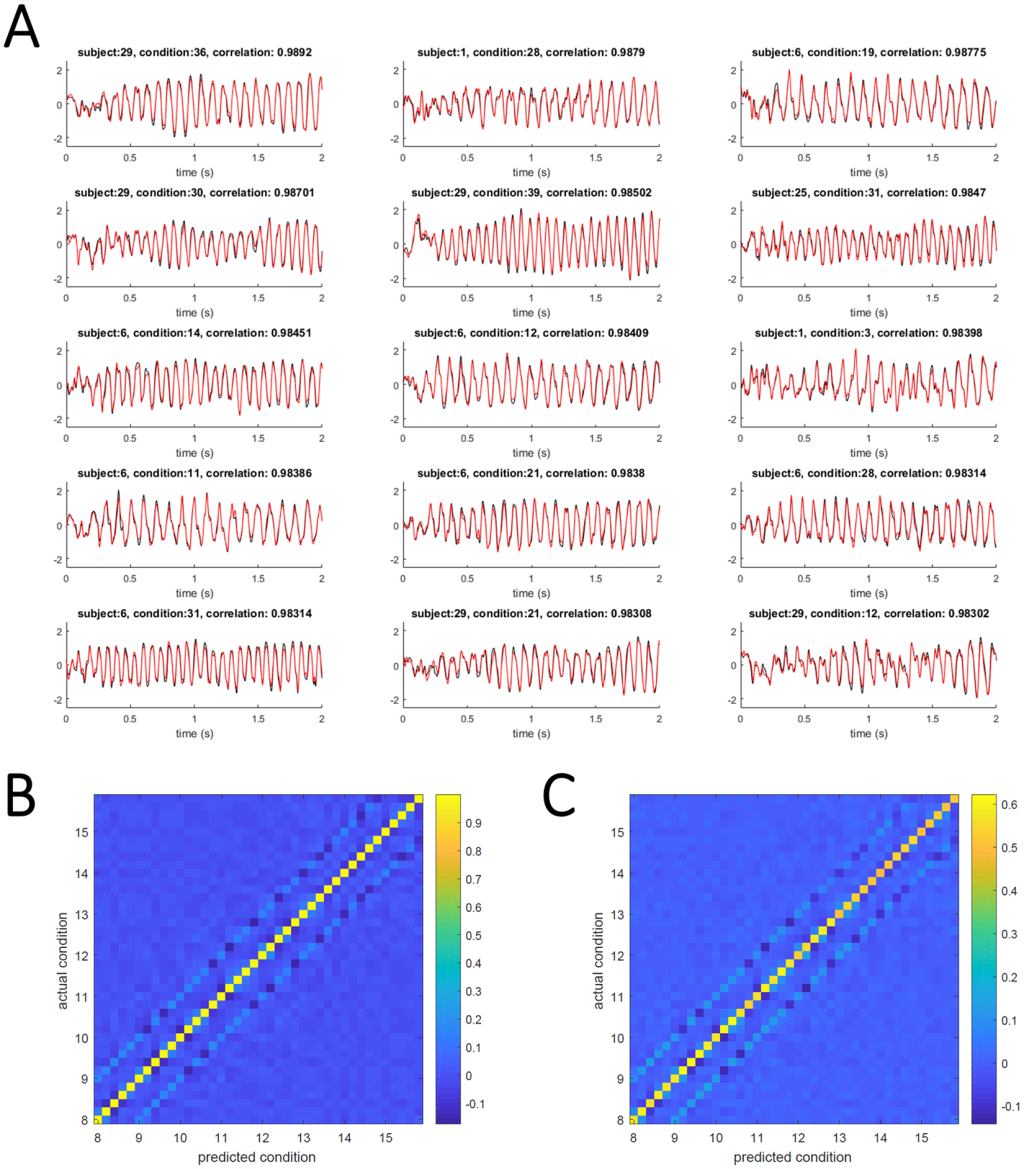
Results of predictive filter. (**A**) Task-related components computed from filters constructed from full data (black traces) and predictive filters (red traces). Here, eight exemplar TRCs that had maximal correlation coefficients are included. Condition-by-condition summary of correlation coefficients between trial-reproducible components constructed (**B**) from full data and (**C**) from predictive filters.

Next, we investigated how well spatial filters trained with data of existing group of subjects could predict a spatial filter of a new subject whose data was not used for training. As previous studies on SSVEP-based BCIs have demonstrated robust SSVEPs among subjects, SSVEPs are appropriate to corroborate the proposed method. Because the SSVEP dataset had a fixed number of subjects, one subject out of 35 subjects was chosen as “a new subject,” and the data from the other subjects were used for training. Figure 7 summarizes the results of predicted TRCs. Figure 7A compares the TRCs computed with full data set (see *Group-level trial-reproducibility maximization (gTRCA) algorithm*) (black lines) and the TRCs computed with only one trial data of the new subject (see *Predictive filter computed from group data*) (red lines). Clearly, the predicted TRCs closely matched the TRCs computed with the full data set. To see the similarity and dissimilarity of those TRCs across conditions, correlation matrices were computed. The TRCs computed from the full data set led to a correlation matrix with approximately diagonal structure (Fig. 7B), suggesting that a TRC derived in one condition was not correlated with TRCs derived in the other conditions. Essentially, the same diagonal structure was observed in the correlation matrix of predicted TRCs (Fig. 7C). This result confirms that the proposed method of predictive filter successfully generalized the existing set of filters to a new subject.

## Discussion

As an extension of our previous method (TRCA, Fig. 1), this study proposed a signal processing method (group-level TRCA) that extracts task-related EEG components across all trials, sessions, and subjects (Fig. 2). gTRCA was successfully applied to and validated with the SSVEP dataset of 35 subjects and was shown to be able to extract the reproducible components time-locked to the visual flickering stimuli in comparison with single-channel analysis or SSD (Figs. 3 and 4). We stress that gTRCA outperformed SSD in extracting the frequency components, even though gTRCA required only trial onsets as input parameter whereas SSD needed additionally a desired frequency band for enhancement and its neighboring bands for suppression. The corresponding scalp maps obtained by gTRCA were more reproducible across both conditions and subjects than those obtained by SSD (Fig. 5). It should be noted that the reproducibility of the scalp maps was non-trivial because gTRCA maximizes only the reproducibility in the time domain, indicating the temporally reproducible components have corresponding anatomical loci common to subjects. The reproducibility of task-related components was confirmed by mapping the single-trial time series into the two dimensions, and we found that, in comparison with TRCA, the reproducibility of gTRCA originated from the inter-subject similarity maximization (Fig. 6). Furthermore, gTRCA could construct a predictive filter even when one trial data of a new subject is provided by exploiting the similarity with trial-reproducible components of existing subjects used for training (Fig. 7). We believe that gTRCA allows to obtain a robust result against within- and across-subject variability, which should be a useful not only for conventional EEG research but also for BCI research to overcome intra- and inter-subject variability. Here we branch out to discuss some implications and applications of trial-reproducibility maximization.

### Previous approaches to multivariate ERP analyses

In line with gTRCA, there have been approaches based on multivariate signal processing to enhance and decompose ERPs. Particularly, approaches based on principal component analysis and its variants have been proposed and widely applied to various ERP components^6,10,11,41,42^. PCA is applied to either the spatial domain (e.g., electrodes), the time domain (e.g., time series), the frequency domain (e.g., Fourier coefficients), or the time-frequency domain (e.g., wavelet transform). The PCA-based ERP analyses have been successful in extracting ERP components that are interpretable as certain cognitive processes^7–10^. Spatial and temporal PCA, for example, have been employed to an oddball experiment and separated a P300 component with a posterior topography associated with rare and task-related tones and a novelty P3 component with a frontal topography associated with novel and task-unrelated tones^43^. Furthermore, to separate temporally overlapping but spectrally segregated components, PCA was applied to time-frequency responses^3–5^. Various ERP responses are not broadly distributed in the frequency domain but have their own spectra; for example, P300 are in the delta band whereas feedback-related negativity are in the theta band^4^. PCA automatically decomposes a time-frequency response into spectrally distinct components, thereby overcoming overlapping in the time domain. Related to the PCA-based approach, a multivariate partial-least-squares (PLS) analysis has been proposed to identify an experimental difference in ERP data^12^. A difference between experimental conditions was explicitly modeled as a design matrix, and components were identified using singular value decomposition. ERP waveforms corresponding to the difference between behavioral conditions (hit and correct rejection trials) were expressed at multiple timepoints and electrodes.

It is known that factor analysis has inherent indeterminacy of a loading matrix up to an orthogonal transformation and that a loading matrix is not uniquely determined. Therefore, for typical neuroscientific or psychological applications, the rotation indeterminacy needs to be resolved by imposing an additional rotation procedure according to a certain optimization criterion. Several such rotation procedures have been proposed including Varimax^44^, Promax^45^, Oblimin^46,47^, and Geomean^48^ rotations, and comparison studies provide practical recommendations in applying these rotation methods to ERP analyses^6,49^. We note that, in contrast, TRCA has no rotation indeterminacy as observed below. The generalized eigenvalue problem of TRCA is ${\bf{S}}{\bf{W}}={\bf{Q}}{\bf{W}}{\boldsymbol{\Lambda }}$ where ${\boldsymbol{\Lambda }}={\rm{diag}}({\lambda }_{1},\cdots ,{\lambda }_{N})$. Let us suppose that the matrix **W** is a solution of this generalized eigenvalue problem and introduce another rotated matrix ${\bf{W}}^{\prime} \equiv {\bf{W}}{\bf{R}}$ with any orthogonal matrix **R**. Clearly, the rotated matrix ${\bf{W}}^{\prime}$ is not a solution of the eigenvalue problem, so a solution of TRCA is unique.

In addition to the PCA-based approach reviewed above, a multivariate approach based on component reproducibility has attracted recent attention^15–19,23,50^. The reproducibility-based approach has increasingly been applied to analyses of functionally meaningful components of EEG responses to finger movements^15^, working memory^16^, visual stimuli^18^, oddball stimuli^17^, tones and natural sounds^29,31^, movies viewing^19^, and perceptual decisions^50^. Up to this point, the reproducibility-based approach is formulated in terms of either inter-trial reproducibility or inter-subject similarity. On one hand, inter-trial reproducibility within individual subjects is maximized in various formulations such as denoising source separation^29,30^, xDAWN^23,29^, reliable component analysis^18,19^, and joint decorrelation^31^. On the other hand, inter-subject similarity among a group of subjects is maximized in formulations such as correlated component analysis^19^ and multiway canonical correlation^51^. The gTRCA algorithm developed in this study naturally extends the previous studies by combining inter-trial reproducibility and inter-subject similarity in a single objective function (Eq. (12)). Alternatively, it is possible to perform a two-step analysis in which maximization of inter-trial reproducibility within individuals is followed by maximization of inter-subject similarity across a group of subjects. The single-step approach like ours is conceptually and algorithmically simple, yet the two-step approach may be more flexible if additional process of dimensional reduction is applied before proceeding to maximization of inter-subject similarity.

### Implications on group-level analysis

An issue of conventional two-step approaches (i.e., averaging trials within a subject, then averaging them across subjects) can be typically found in the case of EEG analyses using independent component analysis (ICA)^52^. In the proposed approach, ICA is first applied to individual data to decompose scalp-recorded signals into temporally maximally independent components. However, ICA reveals individual differences and results in different number of ICs in different locations from subject to subject, which prevents us from simple averaging, so grand mean of independence components is no longer available. To address the group-level inconsistency across subjects, they perform unsupervised clustering on features of ICs from all the subjects. The problem is that this clustering process needs inputs that are not obviously determined. One input is how to determine a feature vector corresponding to an IC. A proposal is that a feature vector includes an equivalent dipole location, an ERP waveform and a spectrum. However, there are a number of options regarding how to define a feature vector, and the choice of a feature vector would critically influence the results of a group-level analysis. Another input is the number of clusters. If the number of clusters is too small, the diversity of ICs would aggregate and thus disappear. If the number of clusters is too large, one cluster would contain only a handful of components, and that cluster would not represent a typical EEG component. Therefore, the two-step approaches reviewed above are plagued by the choices of feature vectors and the number of clusters.

On the other hand, the proposed gTRCA algorithm is free from the problem of arbitrary parameters and is a natural formulation of finding a common and reproducible component in a group of subjects. One of the goals in a group-level analysis is to suppress effects of intra- and inter-subject variability and enhance a task-related component that is reproducible and common and to all subjects. Maximizing trial reproducibility within and across individual subjects is hence a direct way to achieve the goal of a group-level analysis. In the gTRCA algorithm, only timings of trial onsets and a trial duration are required to specify, which are naturally determined by experimental conditions. There are no other arbitrary parameters. Therefore, we believe that gTRCA provides another method to complement the grand-averaging group analysis and the ICA-based group analysis for epoched data.

Finally, we note an important limitation of TRCA and its extensions in comparison PCA- or ICA-based approaches. TRCA is applicable only to epoched data segmented with trial time windows but not to continuous data, whereas PCA- and ICA-based approaches can handle both epoched and continuous data. Accordingly, TRCA and its extensions cannot be employed for a group analysis of continuous data. One notable example of such continuous data is those of spontaneous activity in resting-state experiment; ICA has been successfully applied to continuous data to discover functionally interpretable components^53–55^. We recommend the potential users to understand the advantage and the limitation of TRCA and to apply TRCA and other methods in a complementary manner.

### Three approaches: hypothesis-driven, data-driven, and guided

Broadly speaking, there are two widely practiced approaches to neuroimaging data analysis: hypothesis-driven and data-driven approaches^56,57^. The hypothesis-driven approach — the best example can be found in general linear model (GLM) analyses^58^ — posits experiment-specific hypotheses that certain effects of experimental conditions be reflected in certain brain activities. The hypotheses are typically formulated in terms of a design matrix predicting experimental effects as a time courses, which are correlated with neuroimaging data. On the other hand, the data-driven approach — a good example here is the use of independent component analysis (ICA)^59,60^ — posits only general assumptions about data, such as mutual independence of sources, and does not require any assumptions specific to the experimental effect of interest. Although both of these approaches have been widely and successfully used in neuroscience researches, they still have limitations. For example, in the hypothesis-driven approach, prediction of the time-course of the targeted neural activity is not always obvious, particularly for EEG whose event-related responses are heterogeneous, on which simple modeling does not work. Besides, those effects that are not modeled in a design matrix will not be detectable by definition. In the data-driven approach, a result from the analysis requires interpretations by analysts to determine whether the obtained component is a signal or noise, and if it is a signal, what it signals is and what it means. So, it depends on interpretations, with which the result become meaningful and available for scientific discussions. The process of interpretation, however, depends on subjective judgements and analyst’s personal experiences. It is a valid approach on its own (see clinical researcher for example for how they handle these issues), but objectivity and reproducibility remain to be a challenge.

In contrast to the two approaches seen above, yet another approach in neuroscientific data analyses is to make active use of prior knowledge about data or experimental conditions—namely, a guided approach. For example, it is known that a specific frequency range of EEG data is correlated with certain cognitive functions and/or experimental conditions, and also that oscillatory responses, including SSVEPs and motor µ rhythms, have been widely analyzed in extant researches. Then, linear spatial filters may be constructed to maximize the signal-to-noise ratio in a frequency range of interest against the surrounding frequency ranges (for example, see spatio-spectral decomposition^39^ and joint decorrelation^31^). Another example of the guided approach is to construct a linear spatial filter that maximizes the variance in one condition while suppresses the variance in another condition, often known as common spatial filter. It is noteworthy that many of guided approach methods are formulated in the framework of generalized eigenvalue problems. The guided approach incorporates our general knowledge about neuroscientific data and experimental conditions to an analysis but does not require specific predictions on activation time series and relevant brain areas. We cast our TRCA algorithm into the category of the guided approach; the only general assumption is that there be TRCs within and across subjects. Thus, gTRCA requires only the timings of trial onsets and the trial duration. We believe that the guided approach provides a complementary method for analyzing EEG data which are by nature variable across conditions and subjects.

### Application to brain-computer interfaces

The reproducibility-based approach such as TRCA and correlated component analysis has attracted recent attention in the field of brain computer interfaces^61–64^. Inherent non-stationarity and variability of EEG have been an impediment in developing robust and training-free BCIs^33,65^. A typical problem is that a classifier trained with data from a certain session/subject may not generalize well to a new session/subject. For example, intersession non-stationarity is caused by varying impedances and positions of electrodes from session to session (and the same happens across subjects as well). Due to intersession non-stationarity, a classifier needs to be trained at the beginning of every session before use, making an application of BCIs to daily use laborious not impossible. Thus, for the best performance a classifier needs to be trained every time new data become available. In contrast, zero-training BCIs using transfer learning benefits from using other sessions/subjects for learning so that it does not need to re-learn the data from scratch every time training data are updated^32,66^. Our proposed method shares the idea with zero-training BCIs, and we demonstrated that the computation of gTRCA can be straightforwardly applied to a new subject given the dataset of exiting subjects. Therefore, a gTRCA-based BCI allows an online application from the beginning of epoch.

SSVEP has been intensively studied for applications to brain-computer interfaces^67–70^. Recently, a TRCA-based SSVEP speller that was based on individual subjects was shown to outperform other methods^61,62^. They defined and extracted TRCs within single subjects as the original TRCA was formulated. As shown in the main text, incorporating the inter-subject reproducibility enhanced the degree of clustering of task-related components (Fig. 6). It is a promising future direction to investigate whether and how the gTRCA algorithm enhances the performance and the inter-subject generalization of an SSVEP speller. Particularly, the predictive filter for a new subject allows for online application from the beginning of the epoch. Up to this point, applications of TRCA-based methods have focused on SSVEP-based BCIs. Our recent study demonstrated that a variant of TRCA (cross-correlation TRCA or xTRCA) serves a preprocessing step in enhancing the discriminability of ERPs from standard and deviant conditions in a mismatch-negativity experiment^17^. The PCA-based method reviewed above is sensitive to a reduced signal-to-noise ratio, and it should be investigated how robust the reproducibility-based approach is under various noise conditions. We hope that applicability and robustness of reproducibility-based approach for BCI applications will be explored in future studies.

### Conclusion

We proposed and demonstrated a group-level trial-reproducibility maximization that suppresses effects of non*-*interest across sessions/subjects more actively and effectively than conventional univariate and multivariate methods. We conclude that gTRCA offers a useful framework for group-level EEG data analysis for understanding a common neural processing of cognitive function and for constructing robust and training-free brain-computer interfaces.

## Supplementary information

Supplementary Info.

Dataset 1.

## Data Availability

Supplementary Materials describe Matlab demo scripts, functions, sample data for group TRCA, and additional analyses. These files are included as Supplementary Data. The full dataset and Matlab scripts are available from the corresponding author on reasonable request.
