# Supplementary material for: Group task-related component analysis (gTRCA): a multivariate method for inter-trial reproducibility and inter-subject similarity maximization for EEG data analysis
Source: Sci Rep. 2020 Jan 9;10:84. doi: 10.1038/s41598-019-56962-2 (PMC6952454; doi:10.1038/s41598-019-56962-2)
Supplement: Supplementary file 1 — Supplementary Info. [file 41598_2019_56962_MOESM1_ESM.pdf]

Group task-related component analysis (gTRCA): a multivariate method for inter-trial reproducibility and inter-subject similarity maximization for EEG data analysis

Hirokazu Tanaka

School of Information Science
Japan Advanced Institute of Science and Technology
1-1 Asahidai, Nomi, Ishikawa 923-1211, Japan

Abbreviated Title: Group task-related component analysis

Type of article: Original research article

Corresponding author:
Hirokazu Tanaka
Email: hirokazu@jaist.ac.jp
Tel: +81-761-51-1226 Fax: +81-761-51-1149

Conflict of interest: The author declares no financial and non-financial competing interests.

Key words: Multivariate analysis; Generalized eigendecomposition; Group-level analysis; Steady-state visual evoked potentials (SSVEPs); Brain-computer interfaces (BCIs)

1. Matlab codes of group task-related component analysis (gTRCA)

Matlab scripts, functions, and sample data are available as Supplementary Data and at the figshare website (https://figshare.com/articles/gTRCA_SciRep_zip/9121751).

This directory contains:

- demo_groupTRCA.m: main script
- groupTRCA.m: matlab function of group TRCA
- blocky.m: matlab function for epoching time series
- topoplotIndie.m: matlab function of plotting a scalp map (courtesy of Dr. Mike X. Cohen)
- multiprod.m: matlab function of multiple matrix multiplication (courtesy of Dr. Paolo de Leva, https://jp.mathworks.com/matlabcentral/fileexchange/8773-multiple-matrix-multiplications-with-array-expansion-enabled)
- 64-channels.mat: data file about channel locations
- Freq_Phase.mat: data file about stimulus frequencies and phases

The data directory contains:

- condition1.mat: 35-subject data from condition 1 (stimulation frequency of 8 Hz)

The sample SSVEP data was created and modified from a subset of the public-domain dataset that is described in the following paper:

Wang, Y., Chen, X., Gao, X., & Gao, S. (2016). A benchmark dataset for SSVEP-based brain–computer interfaces. IEEE Transactions on Neural Systems and Rehabilitation Engineering, 25(10), 1746-1752.

Note that this directory contains only one condition to reduce the file size. In the submitted manuscript, all 40 conditions were analyzed in the same way.

To run the script, type in the Matlab command window:

```
>> demo_groupTRCA
```

The figures of time series and scalp maps of task-related components will appear accordingly (see Supplementary Figures 1 and 2). The attached below is the Matlab function `groupTRCA` used in this study.

```
function [w, d, S, Q] = groupTRCA(X, Xb, tau)

% X: data in cell format X = cell(1, Nsubs)
% X(sub): Nchannels x Nsamples
% Xb(sub): Nchannels x tau x Ntrials

Nsubs = size(X, 2);
[Nchannels, Nsamples] = size(X{1});
Ntrials = size(Xb{1},3);

% computation of U, V, Q0 matrices:
U = zeros(Nchannels, tau, Nsubs);
V = zeros(Nchannels, Nchannels, Nsubs);
Q0 = zeros(Nchannels, Nchannels, Nsubs);

for n=1:Nsubs
    U(:,:,n) = mean(Xb{n},3);
    for k=1:Ntrials
        V(:,:,n) = V(:,:,n) + Xb{n}(:,:,k)*Xb{n}(:,:,k)'/Ntrials;
    end
    Q0(:,:,n) = X{n}*X{n}'/Nsamples;
end

% computation of S and Q matrices:
 S = zeros(Nchannels*Nsubs, Nchannels*Nsubs);
for a=1:Nsubs
    for b=1:Nsubs
        rows= (1+(a-1)*Nchannels):a*Nchannels;
        columns = (1+(b-1)*Nchannels):b*Nchannels;
        if a==b
            Stmp = 2*Ntrials/((Ntrials-1)*tau)*(U(:,:,a)*U(:,:,a)' -
V(:,:,a)/Ntrials);
        else
            Stmp = 1/tau*(U(:,:,a)*U(:,:,b)');
        end
        S(rows, columns) = Stmp;
    end
end
Q = zeros(Nchannels*Nsubs, Nchannels*Nsubs);
for n=1:Nsubs
    rows= (1+(n-1)*Nchannels):n*Nchannels;
    Q(rows, rows) = Q0(:,:,n);
end

% generalized eigendecomposition:
[W, D] = eig(Q\S);
D = diag(D);
[~, index] = sort(D, 'descend');
d = D(index(1));
w = W(:,index(1));
```

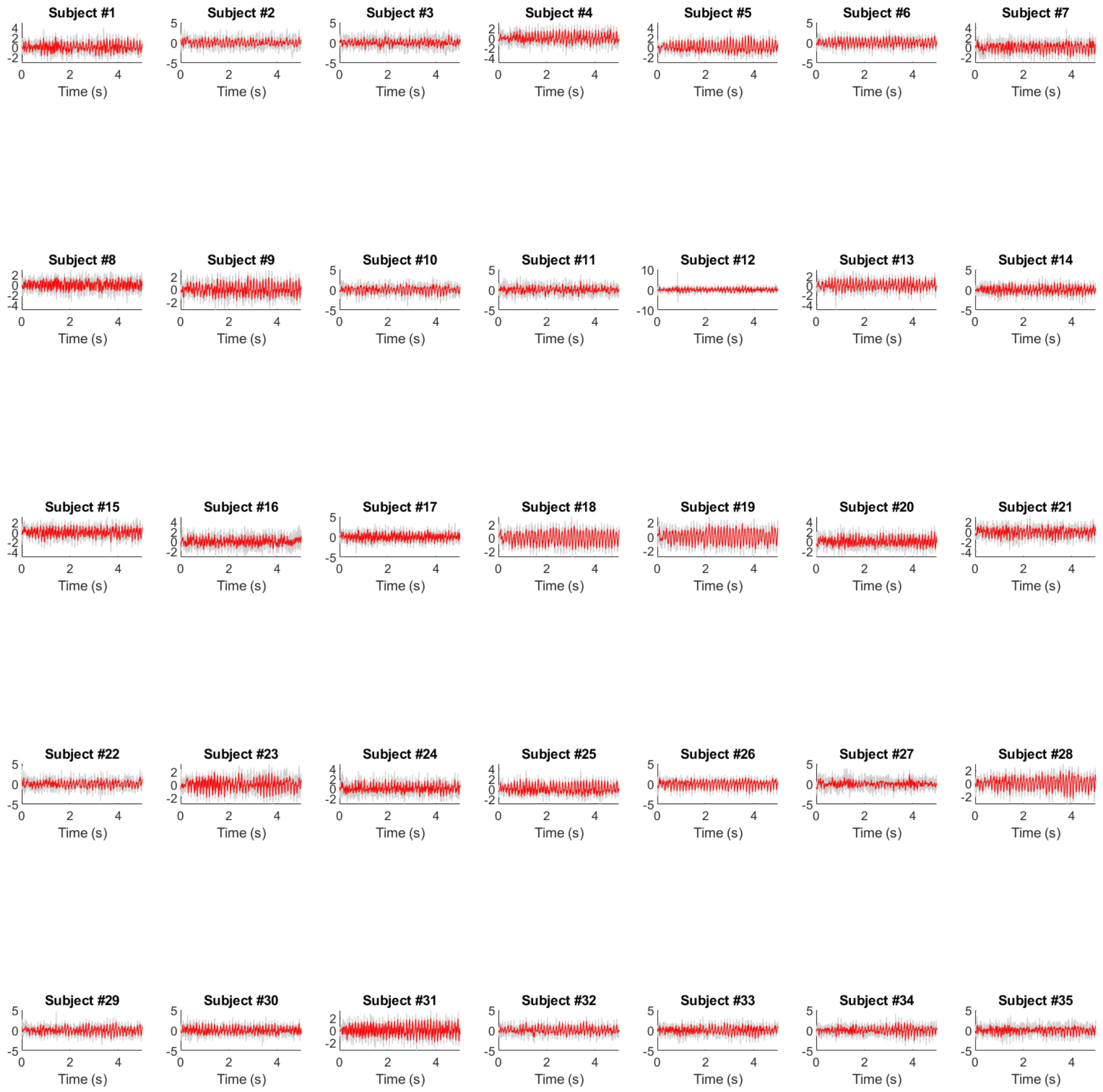

Supplementary Figure 1. Time series of task-related components obtained from group TRCA.

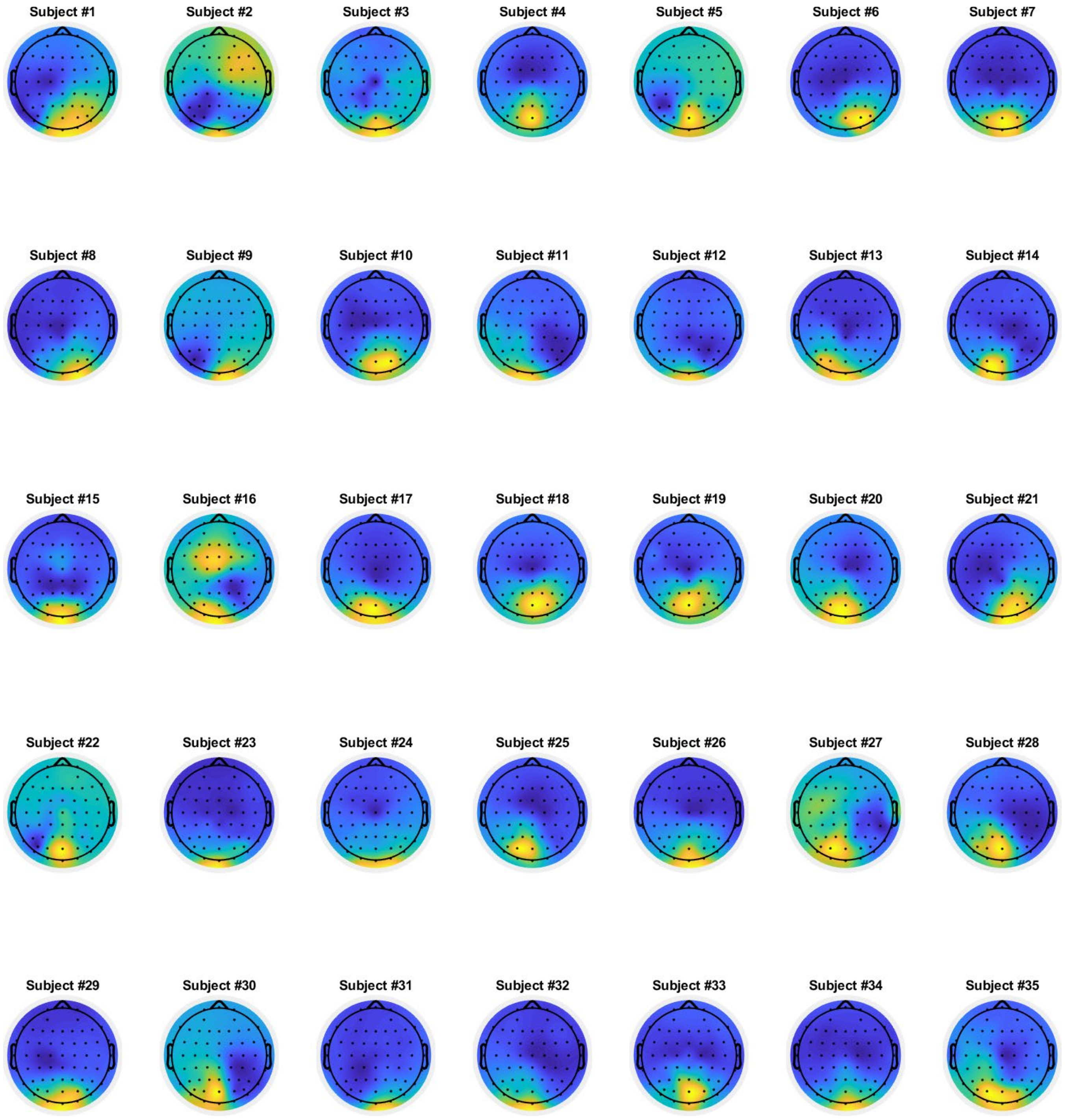

Supplementary Figure 2. Scalp maps corresponding to the task-related components obtained by group TRCA.

2. Supplementary analyses

Here we describe additional analyses to support the results presented in the main text. Specifically, the distributions of eigenvalues, the resampling-based statistical test, and the effect of dimensional reduction are explained.

2.1. Eigenvalue distributions

As described in the main text, the eigenvalues in the generalized eigendecomposition problem measure how corresponding components are reproducible across trials and similar across subjects. We computed the eigenvalues of all the conditions and found that there was a gap between the first dominant and the second dominant eigenvalues (Supplementary Figure 3). This indicates that there was one reproducible component for each condition common to all subjects. Therefore, the component with the dominant eigenvalue was explained in the main text.

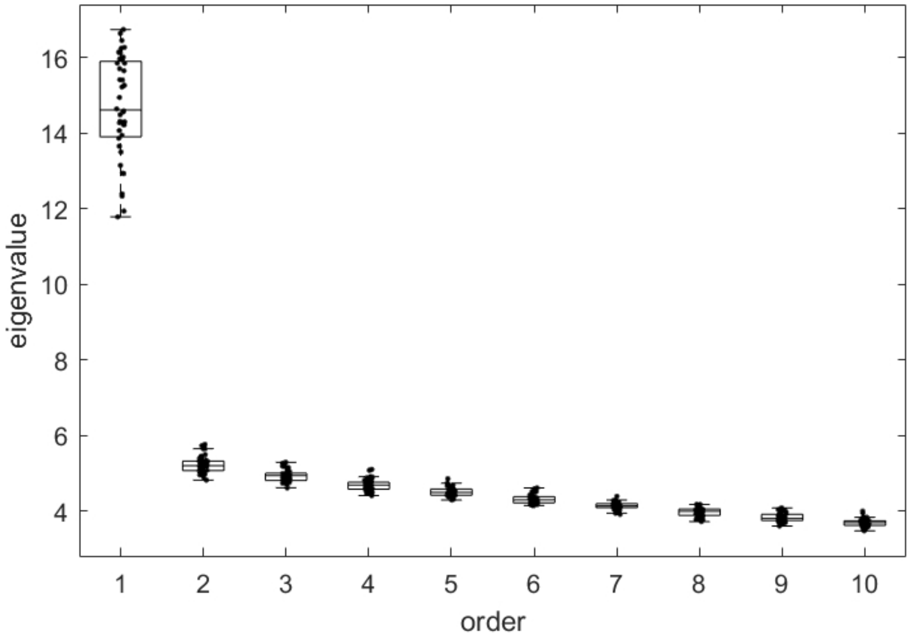

Supplementary Figure 3. Ten dominant eigenvalues for all forty stimulus conditions. Along with the box plots, individual eigenvalues are denoted as black dots. Out of 2,240 eigenvalues, the ten largest eigenvalues are shown for each condition here.

2.2. Resampling-based statistical test

Next, we assessed statistical significance of the dominant components using the resampling-based test proposed in our previous publications (Tanaka, Katura, & Sato, 2013, 2014; Tanaka & Miyakoshi, 2019). The underlying assumption of gTRCA is that there are reproducible signals time-locked to trial onsets. Accordingly, we posit a null hypothesis that there are no reproducible signals time-locked to trial onsets. If the null hypothesis holds, the value of eigenvalue that is computed based on trial timings is statistically indistinguishable from those computed based on randomized timings. Therefore, a null

distribution of largest eigenvalues is computed by sampling a randomized timing of *k*-th trial from a uniform distribution,

$$t_k \sim U\left(0, T-\tau\right),$$

where T is the length of EEG data and τ is the length of time window. Statistical significance of the dominant eigenvalue based on trial timings is tested against the null distribution.

The null distribution of the dominant eigenvalue was computed by resampling randomized trial timings 1,000 times (typically took about 1.5 hours for one stimulus condition). Then, statistical significance of the actual dominant eigenvalue was assessed against the null distribution. As clearly seen in Supplementary Figure 4, the actual dominant eigenvalue was statistically significant for all the conditions ($p<10^{-4}$).

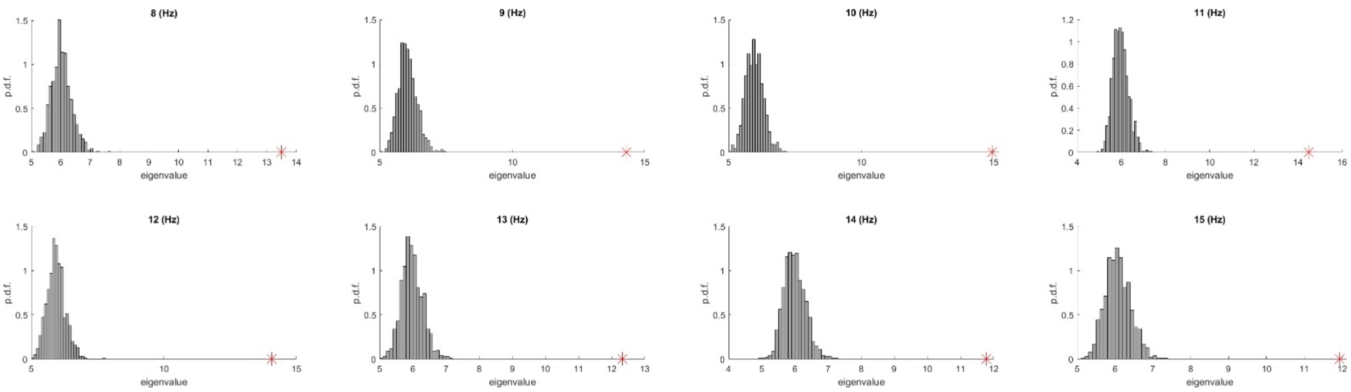

Supplementary Figure 4. Null distributions of maximal eigenvalues (black histograms) and the actual maximal eigenvalues (red asterisks). Note that only the cases of eight stimulation conditions are illustrated here. Similar results were obtained for the other conditions.

2.3. Preprocessing of dimensionality reduction

It is often a common practice to apply a dimensional reduction method as a preprocessing before proceeding to a further analysis (de Cheveigne et al., 2019; de Cheveigne & Simon, 2008). Principal component analysis (PCA) is a standard choice for such a method for dimensional reduction. The SSVEP dataset used in this study contains a relatively large number of electrodes (i.e., 64 channels) and subjects (35 subjects). gTRCA hence optimizes 2,240 (=64×35) weight coefficients. We set out to examine whether a preprocessing of dimensional reduction benefited the results of gTRCA.

Individual data were first subjected to PCA. We found that, in most subjects, about 30 PCs were sufficient to explain 90% of the original variance, so data from individual subjects were reduced to the space of 30 PCs. The dimensionally-reduced data were then analyzed using the gTRCA algorithm. The scalp maps of the dominant component were constructed by projecting from the space spanned by the 30 PCs to the space spanned by the 64 electrodes. The time courses and the scalp maps obtained from the dimensionally-reduced dataset were compared with those obtained from the original dataset (those shown in Supplementary Figure 1). Both the time series and the scalp maps were highly similar

(correlation coefficient 0.94±0.08 (SD) for the time series, and 0.99±0.07 (SD) for the scalp maps). Therefore, for the case of the SSVEP dataset, the effect of PCA preprocessing was not significant.

3. Equivalence between xDAWN and TRCA algorithms

Here we show that, despite the seemingly different formulations, the TRCA and xDAWN algorithms are equivalent under certain conditions. The xDAWN algorithm was proposed to enhance evoked potentials by assuming a generative model that the continuous data $\mathbf{X} \in \mathbb{R}^{n \times T}$ is generated a product $\mathbf{AD}$ where $\mathbf{A} \in \mathbb{R}^{n \times \tau}$ is a trial-reproducible component and $\mathbf{D} \in \mathbb{R}^{\tau \times T}$ is a Toeplitz matrix. Note that the matrix $\mathbf{D}$ is determined by trial onsets and a trial duration, in the same way as in TRCA. In the xDAWN algorithm, the matrix $\mathbf{A}$ is determined so as to minimize $\|\mathbf{X} - \mathbf{AD}\|_{\mathrm{F}}^2$, thereby leading to $\mathbf{A} = \mathbf{XD}^\top \left(\mathbf{DD}^\top\right)^{-1}$. If there are no overlaps between trials (as in most cases of cognitive and BCI experiments), $\left(\mathbf{DD}^\top\right)^{-1}$ becomes simply $\frac{1}{K}\mathbf{I}$ and the matrix $\mathbf{A}$ is simply the trial average $\mathbf{U}$ in Eq. (4) of the main text. The xDAWN algorithm proposes a spatial filter that maximizes the Rayleigh quotient

$$\frac{\mathbf{w}^\top \mathbf{ADD}^\top \mathbf{A}^\top \mathbf{w}}{\mathbf{w}^\top \mathbf{XX}^\top \mathbf{w}} = \frac{\mathbf{w}^\top \mathbf{UU}^\top \mathbf{w}}{\mathbf{w}^\top \mathbf{XX}^\top \mathbf{w}}.$$

This objective function is equivalent to that of TRCA in Eq. (6) of the main text, except that the numerator of Eq. (6) contains additional term $-\frac{1}{K}\mathbf{V}$, which is negligible when K is large. Therefore, the xDAWN and TRCA algorithms are equivalent provided that trials have no or little overlaps and the number of trials K is large enough. To our knowledge, the xDAWN algorithm has not been extended to a multisubject data set.

References

de Cheveigne, A., Di Liberto, G. M., Arzounian, D., Wong, D. D. E., Hjortkjaer, J., Fuglsang, S., & Parra, L. C. (2019). Multiway canonical correlation analysis of brain data. *NeuroImage, 186*, 728-740. doi:10.1016/j.neuroimage.2018.11.026

de Cheveigne, A., & Simon, J. Z. (2008). Denoising based on spatial filtering. *J Neurosci Methods, 171*(2), 331-339. doi:10.1016/j.jneumeth.2008.03.015

Tanaka, H., Katura, T., & Sato, H. (2013). Task-related component analysis for functional neuroimaging and application to near-infrared spectroscopy data. *NeuroImage, 64*, 308-327. doi:10.1016/j.neuroimage.2012.08.044

Tanaka, H., Katura, T., & Sato, H. (2014). Task-related oxygenation and cerebral blood volume changes estimated from NIRS signals in motor and cognitive tasks. *NeuroImage, 94*, 107-119. doi:10.1016/j.neuroimage.2014.02.036

Tanaka, H., & Miyakoshi, M. (2019). Cross-correlation task-related component analysis (xTRCA) for enhancing evoked and induced responses of event-related potentials. *NeuroImage, 197*, 177-190. doi:10.1016/j.neuroimage.2019.04.049
