# Supplementary material for: Group task-related component analysis (gTRCA): a multivariate method for inter-trial reproducibility and inter-subject similarity maximization for EEG data analysis
Source: Sci Rep. 2020 Jan 9;10:84. doi: 10.1038/s41598-019-56962-2 (PMC6952454; doi:10.1038/s41598-019-56962-2)
Supplement: Supplementary file 2 — Dataset 1. [file 41598_2019_56962_MOESM2_ESM.zip › gTRCA_SciRep/SupplementaryDataTanaka_gTRCA.pdf]

Group task-related component analysis (gTRCA): a multivariate method for inter-trial and inter-subject reproducibility maximization for EEG data analysis

Hirokazu Tanaka

School of Information Science
Japan Advanced Institute of Science and Technology
1-1 Asahidai, Nomi, Ishikawa 923-1211, Japan

Abbreviated Title: Group task-related component analysis

Type of article: Original research article

Corresponding author:
Hirokazu Tanaka
Email: hirokazu@jaist.ac.jp
Tel: +81-761-51-1226 Fax: +81-761-51-1149

Conflict of interest: The author declares no competing interests.

Key words: Multivariate analysis; Generalized eigendecomposition; Group-level analysis; Steady-state visual evoked potentials (SSVEPs); Brain-computer interfaces (BCIs)

Matlab codes of group task-related component analysis (gTRCA)

This directory contains:

- demo_groupTRCA.m: main script
- groupTRCA.m: matlab function of group TRCA
- blocky.m: matlab function for epoching time series
- topoplotIndie.m: matlab function of plotting a scalp map (courtesy of Dr. Mike X. Cohen)
- multiprod.m: matlab function of multiple matrix multiplication (courtesy of Dr. Paolo de Leva, https://jp.mathworks.com/matlabcentral/fileexchange/8773-multiple-matrix-multiplications-with-array-expansion-enabled)
- 64-channels.mat: data file about channel locations
- Freq_Phase.mat: data file about stimulus frequencies and phases

The data directory contains:

- condition1.mat: 35-subject data from condition 1 (stimulation frequency of 8 Hz)

The SSVEP data is created from a subset of the public-domain dataset that is described in the following paper:

Wang, Y., Chen, X., Gao, X., & Gao, S. (2016). A benchmark dataset for SSVEP-based brain–computer interfaces. IEEE Transactions on Neural Systems and Rehabilitation Engineering, 25(10), 1746-1752.

Note that this directory contains only one condition due to reduce the file size. In the submitted manuscript, all 40 conditions were analyzed in the same way.

To run the script, type in the Matlab command window

```
>> demo_groupTRCA
```

The figures of time series and scalp maps of task-related components will appear accordingly (see Supplementary Figures 1 and 2).

```
function [w, d, S, Q] = groupTRCA(X, Xb, tau)

% X: data in cell format X = cell(1, Nsubs)
% X{sub} : Nchannels x Nsamples
% Xb{sub}: Nchannels x tau x Ntrials

Nsubs = size(X, 2);
[Nchannels, Nsamples] = size(X{1});
Ntrials = size(Xb{1},3);

% computation of U, V, Q0 matrices:
U = zeros(Nchannels, tau, Nsubs);
V = zeros(Nchannels, Nchannels, Nsubs);
Q0 = zeros(Nchannels, Nchannels, Nsubs);

for n=1:Nsubs
    U(:,:,n) = mean(Xb{n},3);
    for k=1:Ntrials
        V(:,:,n) = V(:,:,n) + Xb{n}(:,:,k)*Xb{n}(:,:,k)'/Ntrials;
    end
    Q0(:,:,n) = X{n}*X{n}'/Nsamples;
end

% computation of S and Q matrices:
 S = zeros(Nchannels*Nsubs, Nchannels*Nsubs);
for a=1:Nsubs
    for b=1:Nsubs
        rows= (1+(a-1)*Nchannels):a*Nchannels;
        columns = (1+(b-1)*Nchannels):b*Nchannels;
        if a==b
            Stmp = 2*Ntrials/((Ntrials-1)*tau)*(U(:,:,a)*U(:,:,a)' -
V(:,:,a)/Ntrials);
        else
            Stmp = 1/tau*(U(:,:,a)*U(:,:,b)');
        end
        S(rows, columns) = Stmp;
    end
end
Q = zeros(Nchannels*Nsubs, Nchannels*Nsubs);
for n=1:Nsubs
    rows= (1+(n-1)*Nchannels):n*Nchannels;
    Q(rows, rows) = Q0(:,:,n);
end

% generalized eigendecomposition:
[W, D] = eig(Q\S);
D = diag(D);
[~, index] = sort(D, 'descend');
d = D(index(1));
w = W(:,index(1));
```

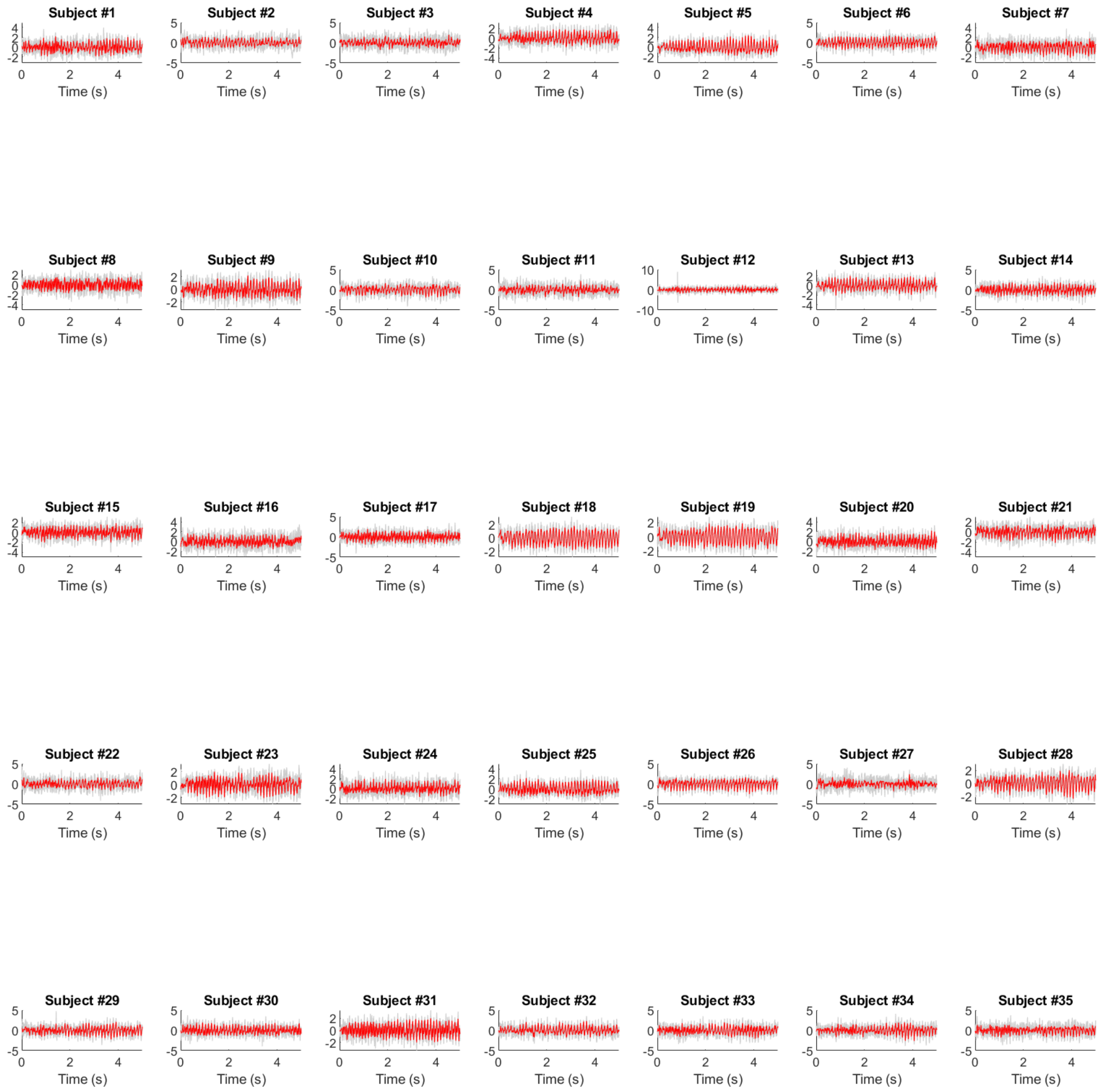

Supplementary Figure 1. Time series of task-related components obtained from group TRCA.

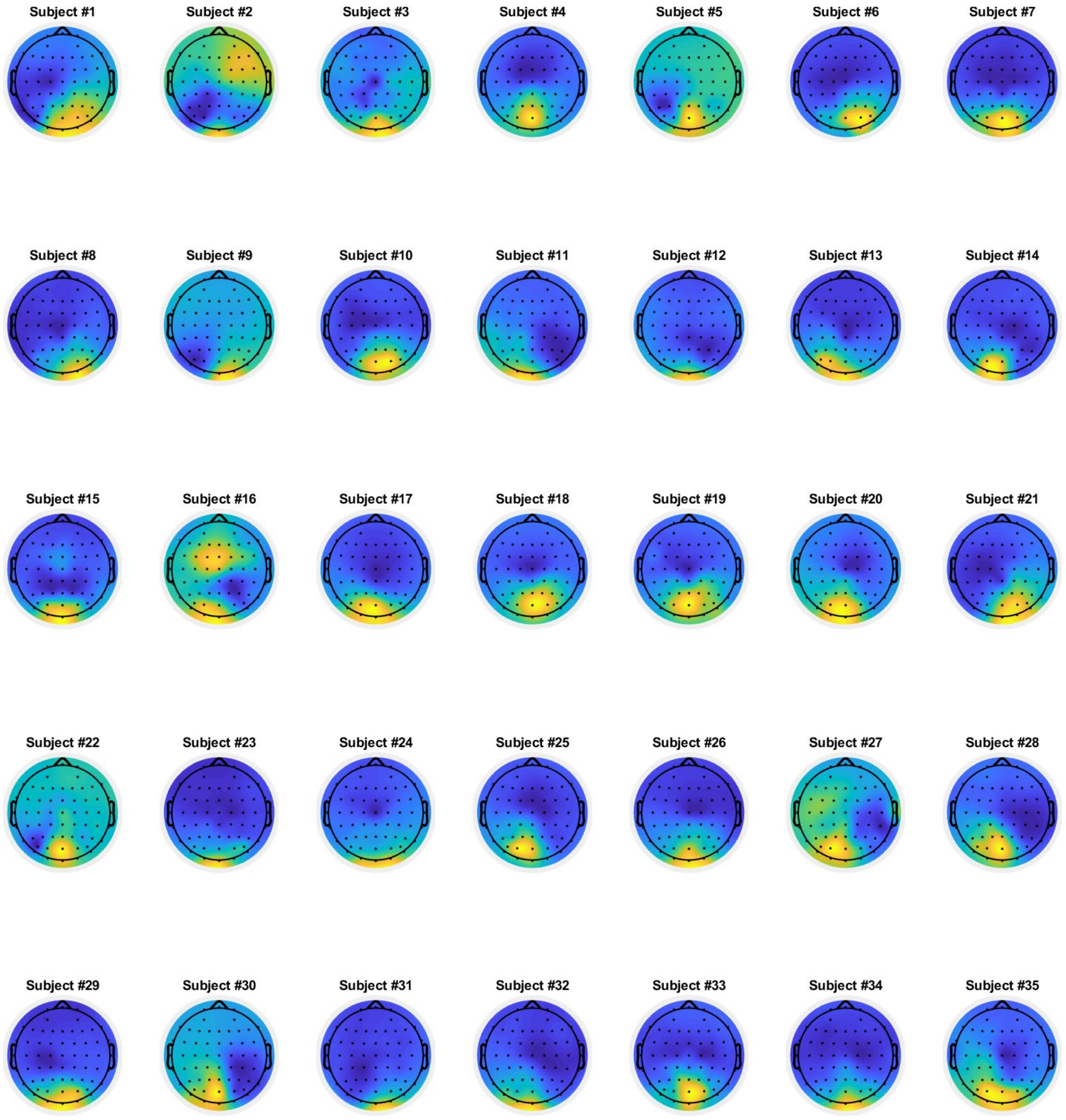

Supplementary Figure 2. Scalp maps corresponding to the task-related components obtained by group TRCA.
